# The *Arabidopsis thaliana* LysM‐containing Receptor‐Like Kinase 2 is required for elicitor‐induced resistance to pathogens

**DOI:** 10.1111/pce.14192

**Published:** 2021-09-30

**Authors:** Moira Giovannoni, Damiano Lironi, Lucia Marti, Chiara Paparella, Valeria Vecchi, Andrea A. Gust, Giulia De Lorenzo, Thorsten Nürnberger, Simone Ferrari

**Affiliations:** ^1^ Dipartimento di Biologia e Biotecnologie “Charles Darwin” Sapienza Università di Roma Rome Italy; ^2^ Department of Plant Biochemistry University of Tübingen, Center for Plant Molecular Biology Tübingen Germany

**Keywords:** *Botrytis cinerea*, chitin, plant innate immunity, priming

## Abstract

In *Arabidopsis thaliana*, perception of chitin from fungal cell walls is mediated by three LysM‐containing Receptor‐Like Kinases (LYKs): CERK1, which is absolutely required for chitin perception, and LYK4 and LYK5, which act redundantly. The role in plant innate immunity of a fourth LYK protein, LYK2, is currently not known. Here we show that CERK1, LYK2 and LYK5 are dispensable for basal susceptibility to *B. cinerea* but are necessary for chitin‐induced resistance to this pathogen. LYK2 is dispensable for chitin perception and early signalling events, though it contributes to callose deposition induced by this elicitor. Notably, *LYK2* is also necessary for enhanced resistance to *B. cinerea* and *Pseudomonas syringae* induced by flagellin and for elicitor‐induced priming of defence gene expression during fungal infection. Consistently, overexpression of *LYK2* enhances resistance to *B. cinerea* and *P. syringae* and results in increased expression of defence‐related genes during fungal infection. LYK2 appears to be required to establish a primed state in plants exposed to biotic elicitors, ensuring a robust resistance to subsequent pathogen infections.

## Abstract 2

*Arabidopsis thaliana* LYK2 is not involved in the direct perception of chitin, but it is necessary for elicitor‐induced resistance to pathogens and priming of defence response.

## 1 INTRODUCTION

The first line of defence that plants use to fend off pathogen attacks relies on pattern recognition receptors (PRRs) on the cell surface that recognize danger signals, called elicitors, that include microbe‐ or pathogen‐associated molecular patterns (MAMPs or PAMPs) and damage‐associated molecular patterns (DAMPs) (Boller & Felix, 2009; Gust, Pruitt, & Nürnberger, 2017). MAMPs are molecules common to all strains of a given taxonomic group of pathogens, such as flagellin, a structural protein of bacterial flagella, and chitin, the major component of fungal cell walls consisting of a polymer of *N*‐acetylglucosamine (GlcNAc) (Boller & Felix, 2009). DAMPs are host‐derived molecules produced during infection (De Lorenzo, Ferrari, Cervone, & Okun, 2018), and include, among others, pectin‐derived oligogalacturonides (OGs) (Ferrari et al., 2013), Arabidopsis elicitor peptides (AtPeps) (Bartels et al., 2013) and extracellular ATP (Tanaka, Choi, Cao, & Stacey, 2014). PRR activation triggers, within minutes, very rapid downstream responses, including a transient influx of calcium ions, activation of calcium‐dependent protein kinases, production of reactive oxygen species (ROS), and phosphorylation of mitogen‐activated protein kinases (MAPKs) (Boudsocq et al., 2010; Kadota et al., 2014; Li et al., 2014; Nuhse, Peck, Hirt, & Boller, 2000). These early signalling events are followed by a more delayed induction of responses, including the expression of defence‐related genes, the biosynthesis of antimicrobial compounds and the deposition of callose in the cell wall, resulting in a so‐called pattern‐triggered immunity (PTI) effective against a broad range of pathogens (Boller & Felix, 2009; Macho & Zipfel, 2014; Tang, Wang, & Zhou, 2017).

Well‐characterized plant PRRs are FLAGELLIN SENSITIVE 2 (FLS2) and EF‐Tu RECEPTOR (EFR), that recognize flg22, the elicitor‐active epitope of flagellin, and elf18, the epitope of bacterial Elongation Factor‐Thermo‐unstable (EF‐Tu), respectively (Chinchilla, Bauer, Regenass, Boller, & Felix, 2006; Zipfel et al., 2006). Both FLS2 and EFR are transmembrane receptor‐like kinases (RLKs) with leucine‐rich repeat (LRR) motifs in their extracellular domain. After ligand perception, FLS2 and EFR interact with another LRR‐RLK, BRASSINOSTEROID INSENSITIVE 1‐ASSOCIATED RECEPTOR KINASE 1 (BAK1), which induces downstream responses (Chinchilla et al., 2007). Another important class of PRRs are RLKs containing three Lysin motifs (LysMs) in their ectodomain (LysM‐RLKs, or LYKs) (Buendia, Girardin, Wang, Cottret, & Lefebvre, 2018). GPI‐anchored LysM‐containing receptor‐like proteins (LYPs) lacking an intracellular kinase domain are also found in plants (Arrighi et al., 2006). Most LYKs and LYPs that have been studied perceive structurally related GlcNAc‐containing molecules and are involved in plant‐microbe interactions (Buendia et al., 2018). The first identified LYKs were Nod Factor Receptor 1 (LjNFR1) and 5 (LjNFR5) of *Lotus japonicus* (Limpens et al., 2003; Radutoiu et al., 2007), and MtLYK3 and MtLYK4 of *Medicago truncatula* (Limpens et al., 2003), that act as receptors of Nod factors, lipochitooligosaccarides (LCOs) produced by nitrogen‐fixing rhizobia. While some LYKs, like LjNFR1 and MtLYK3, have a canonical RD kinase and show in vitro autophosphorylation, others carry an aberrant kinase domain lacking some conserved features and do not exhibit either auto‐phosphorylation or trans‐phosphorylation activities in vitro, indicating that they require one or more co‐receptors to exert their function (Arrighi et al., 2006; Klaus‐Heisen et al., 2011; Madsen et al., 2011).

Following the identification of LYKs involved in LCO perception, OsCEBiP, a LYP lacking an intracellular kinase domain, was found to be the rice receptor for chitin (Kaku et al., 2006). OsCEBiP acts in cooperation with the LYK co‐receptor OsCERK1, that is essential for chitin signalling in rice (Shimizu et al., 2010). The Arabidopsis genome encodes five predicted LYKs: AtCERK1/LysM‐RLK1 (henceforth, CERK1) and AtLYK2 to AtLYK5 (henceforth, LYK2 to LYK5), of which only CERK1 and LYK3 are predicted to possess a functional kinase domain (Tanaka, Nguyen, Liang, Cao, & Stacey, 2012). Recognition of chito‐oligosaccharides (COS) in Arabidopsis is mediated by CERK1, which is absolutely required for chitin perception (Miya et al., 2007; Wan et al., 2008), and by LYK4 and LYK5 (Cao et al., 2014). Direct binding of COS was demonstrated for CERK1 (Iizasa, Mitsutomi, & Nagano, 2010) and LYK5 (Cao, Liang, et al., 2014). LYK5 shows a higher affinity to COS than CERK1 (Cao, Liang, et al., 2014) and, after chitin recognition, interacts with CERK1, which homodimerizes (Liu et al., 2012) and autophosphorylates in a LYK5‐dependent manner (Cao, Liang, et al., 2014). Kinase activity is necessary for CERK1 chitin‐dependent phosphorylation and downstream signalling (Petutschnig, Jones, Serazetdinova, Lipka, & Lipka, 2010). Subsequent to autophosphorylation, CERK1 activates PBL27, a receptor‐like cytosolic kinase that triggers downstream responses (Shinya et al., 2014; Yamada et al., 2016), whereas LYK5 is directed to endocytosis, supposedly to ensure proper receptor turnover (Erwig et al., 2017). LYK4 has a redundant role with LYK5, since *lyk4* and *lyk5* single mutants are still partially responsive to chitin, whereas a double mutant is entirely insensitive to this MAMP (Choi et al., 2014).

Chitin perception is thought to be necessary during fungal infection for proper activation of defences that restrict pathogen invasion, as suggested by genetic evidence. For instance, mutants with defects in *CERK1*, *LYK4* or *LYK5* show increased susceptibility to the fungal pathogen *Alternaria brassicicola* (Cao, Tanaka, Nguyen, & Stacey, 2014; Miya et al., 2007; Wan et al., 2008; Wan et al., 2012), and lack of *CERK1* also enhances susceptibility to *Glovynomices cichoracearum* (Wan et al., 2008), *Plectospherella cucumerina* (Mélida et al., 2018) and *Fusarium oxysporum* f. sp. *cubense* (Huaping, Xiaohui, Lunying, & Unsheng, 2016). In cotton, chitin induces the dimerization and phosphorylation of GhLYK5 and GhLYK1/GhCERK1, contributing to defence against *V. dahlia* and *F. oxysporum* f. sp. *vasinfectum* (Gu, Zavaliev, & Dong, 2017; Wang et al., 2020). Beside their importance in chitin signalling, LYK proteins appear to have additional roles in plant immunity. CERK1, together with two LYPs, LYM1 and LYM3, is required for bacterial peptidoglycan (PGN) perception and basal resistance to *Pseudomonas syringae* pv. *tomato* strain DC3000 (*Pst* DC3000) in Arabidopsis (Willmann et al., 2011). Moreover, CERK1 is involved in the perception of laminarinahexaose and β‐D‐cellobiosyl‐(1,3)‐β‐D‐glucose, two mixed‐link glucans with elicitor activity (Mélida et al., 2018; Rebaque et al., 2021). The role of other LYK proteins is more elusive. In Arabidopsis, LYK3 acts as a negative regulator of basal immunity and a positive regulator of responses to the phytohormone abscisic acid (Paparella, Savatin, Marti, De Lorenzo, & Ferrari, 2014), whereas the function of LYK2 remains to be determined.

Most studies on the role and mode of action of PRRs in plant immunity have focused on responses occurring within minutes or hours upon elicitation (MAPK activation; oxidative burst; early gene expression). However, the establishment of a lasting status of enhanced resistance in response to treatments with MAMPs or DAMPs likely depends on long‐term responses that are at least partially dependent on the accumulation of phytohormones, such as salicylic acid (SA), ethylene and jasmonates (Berens, Berry, Mine, Argueso, & Tsuda, 2017; Broekgaarden, Caarls, Vos, Pieterse, & Van Wees, 2015; De Vleesschauwer, Xu, & Höfte, 2014; Wasternack, 2007). For instance, resistance against the necrotrophic fungus *Botrytis cinerea* induced in Arabidopsis by pre‐treatments with flg22 or OGs requires the biosynthesis of camalexin (Ferrari et al., 2007) and an intact ethylene signalling pathway (Gravino, Savatin, Macone, & De Lorenzo, 2015).

Inducible defences are costly, and their improper activation might reduce plant fitness (Huot, Yao, Montgomery, & He, 2014). Plants have therefore evolved the ability to acquire a pre‐conditioned state of defence after specific stimulation by microbial infections or environmental stresses. Exposure to MAMPs or DAMPs might increase the activation of defence responses upon subsequent perception of the same or a different elicitor, ensuring a robust resistance with a low fitness cost. However, the relative contribution of the perception and signalling mediated by specific PRRs in elicitor‐induced resistance is not well understood. In this work, we have investigated the role of LYK proteins in Arabidopsis basal and elicitor‐induced resistance to pathogens. Our results indicate that basal resistance to *B. cinerea* does not require an intact chitin perception system, but resistance to this pathogen induced by exogenous chitin is impaired by mutations in *CERK1*, *LYK2* and *LYK5*. Notably, *LYK2* is required for enhanced resistance to fungal infection observed after pre‐treatments with different elicitors and contributes to basal and induced resistance to *Pst* DC3000. Our results suggest that LYK2 is largely dispensable for chitin perception and early signalling and has a more general role in the regulation of elicitor‐induced priming of defence responses during pathogen infection.

## 2 MATERIALS AND METHODS

### 2.1 Plant material and growth conditions

*Arabidopsis thaliana* (L.) Heynh plants used in this work were all in the Columbia‐0 (Col‐0) background. Seeds of *cerk1‐2* (Miya et al., 2007) were a kind gift of Dr. Naoto Shibuya (Meiji University, Japan); *lyk5‐2* (Salk_131911C) seeds were kindly provided by Dr Elena Petutschnig (Georg‐August‐University of Göttingen, Germany). The *lyk2‐1* (Salk_152226) and *lyk2‐2* (Salk_012441) lines were obtained from the Nottingham Arabidopsis Stock Centre (NASC) and brought to homozygosity before further characterization. For seedlings assays, Arabidopsis seeds were sterilized in 1.6% sodium hypochlorite and 0.01% sodium dodecyl sulphate (SDS) and stored at 4°C in the dark for 2 days. Then, seeds were evenly distributed on 12‐well‐plates containing 0.5x Murashige‐Skoog (MS) basal salts, 0.5% sucrose, pH 5.6, and transferred to a growth chamber with 12 hr day/12r h night cycle at 22°C with 50% relative humidity. For leaf assays, seeds were distributed in plates containing 0.5x MS basal salts, 0.5% sucrose, 0.7% plant agar, pH 5.6. After sowing, plates were stored at 4°C in the dark for 2 days, then transferred to a growth chamber with 12 hr day/12 hr night cycle at 22°C. Ten‐day‐old seedlings were transferred to soil and grown in the growth chamber under 12 hr day/12 hrnight cycle at 22°C with 50% relative humidity. For co‐immunoprecipitation (Co‐IP) assays *Nicotiana benthamiana* plants were grown at 25°C, 16 hr light, 8 hr dark, 75% relative humidity, for 4–5 weeks. For bimolecular fluorescence complementation (BiFC), *Agrobacterium tumefaciens* (strain GV3101)‐mediated transient expression was conducted on *Nicotiana tabacum* plants cv Petit Havana SR1 grown at 25°C, 16 hr day/8 hr night cycle, 75% relative humidity, for 4–5 weeks.

### 2.2 Mutant genotyping

Genomic DNA was extracted from rosette leaves using the Edwards protocol (Edwards, Johnstone, & Thompson, 1991), using the following extraction buffer: 200 mM Tris–HCl pH 7.5, 250 mM NaCl, 25 mM EDTA, 0.5% SDS. DNA was subjected to PCR using the following primers: CCACATATTTCCGAAGACAAGC, LP_(2–1)_; GTTTCTGCTCTTGATGTTGCC, RP_(2–1)_; GCTTGGACTTTGCACTTTGTC, LP_(2–2)_; AAAGTGTTTGGCTCTCACAGG, RP_(2–2)_; TGGTTCACGTAGTGGGCCATCG, LBa1 (see Figure S1a). PCR products were resolved on 1.5% agarose gel and stained with SYBR™ Safe DNA Gel Stain (Invitrogen).

### 2.3 Elicitors

OGs (DP 10–15) were obtained as previously described (Pontiggia et al., 2015). Colloidal chitin was obtained by thoroughly grinding shrimp shell chitin (Sigma Aldrich) with a pestle in a tube containing sterile milliQ water. Flg22 (QRLSTGSRINSAKDDAAGLQIA) was synthesized by EZBiolab (Carmel, IN, USA).

### 2.4 Pathogen growth and infection

*Botrytis cinerea* (Ferrari, Plotnikova, De Lorenzo, & Ausubel, 2003) was grown for 10–15 days at 22°C on MEP medium [malt‐agar 2% (w/v), peptone 1% (w/v) and micro‐agar 1.5% (w/v)] until sporulation. Before plant inoculation, spores were suspended at a final concentration of 5 × 10^5^ conidiospores ml^−1^ in 24 g l^−1^ potato dextrose broth (Difco, Detroit, USA) and incubated for 2–3 hr at room temperature (RT). Four‐week‐old Arabidopsis plants were inoculated placing 5 μl drops of the spore suspension on each side of the middle vein of fully expanded rosette leaves. Plants were covered with a clear plastic dome to ensure high humidity and incubated at 22°C with a 12 hr photoperiod. Lesion areas were determined 48 hr postinfection by measuring water‐soaked lesions, using ImageJ software (https://imagej.nih.gov/ij/). For elicitor‐induced protection, plants were sprayed with water, 200 μg ml^−1^ OGs, 1 μM flg22 or 100 μg ml^−1^ colloidal chitin 24 hr before inoculation, as previously described (Ferrari et al., 2007).

*Pst* DC3000 was inoculated in LB liquid medium containing 25 μg ml^−1^ rifampicin and grown under agitation (200 rpm) for 8–12 hr at 28°C, until OD_600_ = 0.6–1.0. Fully expanded rosette leaves of four‐week‐old plants were syringe‐infiltrated with a suspension of bacteria [1 × 10^6^ colony‐forming units (cfu) ml^−1^] as previously described (Katagiri, Thilmony, & He, 2002). Leaf discs were collected right after inoculation, 2 and 3 days after infiltration, and ground in water to collect bacteria. For each sample, a 1:10 dilution series was plated on solid LB medium containing rifampicin, and colonies were counted after incubation at 28°C for approximately 3 days.

### 2.5 Determination of MAPK phosphorylation

Seedlings (about 100 mg) were homogenized in 100 μl extraction buffer [50 mM Tris–HCl pH 7.5, 200 mM NaCl, 1 mM EDTA, 10 mM NaF, 2 mM sodium orthovanadate, 1 mM sodium molybdate, 10% (v/v) glycerol, 0.1% (v/v) Tween‐20, 1 mM 1,4‐dithiothreitol, 1 mM phenylmethylsulfonyl fluoride, phosphatase inhibitor cocktail P9599 (Sigma, MO)]. Total protein extracts were quantified with Bradford assay (Bio‐Rad). Equal amounts of proteins were separated on 8% polyacrylamide (30% acrylamide/Bis solution, 29:1, Bio‐Rad) SDS gel. Proteins were transferred to a nitrocellulose membrane using TransBlot Turbo (Bio‐Rad). The filter was stained for 10 min with Ponceau‐S Red (Sigma Aldrich) to assess equal loading and then blocked with 5% (w/v) bovine serum albumin (BSA, Sigma Aldrich) in Tris‐Buffered Saline containing 0.1% (v/v) Tween‐20 (TBS‐T; Bio‐Rad) for 2 hr at RT. Membranes were then incubated overnight in TBS‐T containing 0.5% (w/v) BSA and primary antibodies against phospho‐p44/p42 (1:2500) (Cell Signalling Technologies) or MPK3 (1:2500) and MPK6 (1:10000) (Sigma‐Aldrich). Membranes were then incubated with horseradish peroxidase‐conjugated anti‐rabbit antibody (GE‐Healthcare) diluted at 1:6000 in TBS‐T with 0.5% (w/v) BSA. Signal detection was performed using Clarity™ Western ECL substrate detection kit (Bio‐Rad) and a ChemiDoc MP imaging system (Bio‐Rad). As controls, primary antibodies against actin (Sigma) were used.

### 2.6 Oxidative burst assays

Hydrogen peroxide production was measured by a luminol‐based assay as previously described (Galletti, Ferrari, & De Lorenzo, 2011). Leaf discs (0.2 cm^2^) from four‐week‐old plants were washed for 2 hr with water and incubated overnight in a 96‐well plate (one disc per well). Water was then replaced with a solution of luminol (Sigma‐Aldrich; 30 mg ml^−1^) and horseradish peroxidase (Sigma‐Aldrich; 20 mg ml^−1^) containing 100 nM flg22. For chitin elicitation (100 μg ml^−1^), discs were vacuum infiltrated with the chitin solution for 2 min before the addition of the luminol/peroxidase solution. Plates were analysed for 40 min using a GloMax 96 microplate luminometer (Promega) and a signal integration time of 1 s. Luminescence was expressed in relative light units (RLUs).

### 2.7 Callose deposition assays

Rosette leaves of four‐week‐old plants were syringe‐infiltrated with chitin (100 μg ml^−1^), flg22 (100 nM) or water as control. After 24 hr, 10 leaves from at least four independent plants for each treatment were cleared and dehydrated with 100% (v/v) boiling ethanol. Leaves were fixed in an acetic acid: ethanol (1:3) solution for 2 hr, sequentially incubated for 15 min in 75% (v/v) ethanol, 15 min in 50% (v/v) ethanol, 15 min in 150 mM phosphate buffer pH 8.0, and then stained in 150 mM phosphate buffer pH 8.0, containing 0.01% (w/v) aniline blue for 16 hr at 4°C. After staining, leaves were mounted in 50% (v/v) glycerol and examined by a UV epifluorescence microscope (Nikon, Eclipse E200) equipped with a cooled charge‐coupled device camera (DS‐Fi1C). Images were acquired with the Nis Elements AR software (Nikon). Fluorescence intensity in each image was calculated using ImageJ (https://imagej.nih.gov/ij/).

### 2.8 Gene expression analysis

Total RNA was extracted using RNA isolation NucleoZol (Macherey‐Nagel) according to the manufacturer's instructions and treated with Turbo‐DNase I (Ambion). cDNA was synthesized with ImProm‐II™ Reverse Transcription System (Promega). qRT‐PCR was performed with a CFX96 Real‐Time PCR System (BioRad) using SYBR Green Real‐Time PCR Master Mix (Promega) as recommended by the manufacturer. The amplification protocol consisted of 30 s of initial denaturation at 95°C, followed by 45 cycles of 95°C for 15 s, 58°C for 15 s and 72°C for 15 s. Melting curves were recorded to verify single product amplification. For each experiment, dilution series of pooled cDNA samples were run under the same conditions to calculate primer efficiencies. Gene expression levels were normalized to *UBIQUITIN 5* (*UBQ5*, At4G05320). Three technical replicates were performed for each sample, and data analysis was done, with minor modifications, as previously described (Redwan et al., 2016) using LinRegPCR software (Ruijter et al., 2013). Sequences for all primers used for quantitative PCR and identifiers of the corresponding genes are listed in Table S1. Analysis of the expression of *LYK* genes from publicly available microarray data was performed using Genevestigator (Hruz et al., 2008).

### 2.9 Generation of constructs and transgenic plants

The Red Fluorescent Protein (RFP) coding sequence was amplified by PCR from the pSAT6‐mRFP‐N1 plasmid (Invitrogen) with a high‐fidelity DNA polymerase (Roche), using the following primers: ATCGATCTAGAGTCGACGGTACCG (RFP‐FW) and ATCGAGAGCTCTTAGGCGCCGGTG (RFP‐REV). The PCR product was purified, digested with XbaI and SacI (whose sites were introduced with the PCR reaction) and ligated to a pBI‐121 vector (Invitrogen), after removal of the GUS cassette with XbaI and SacI. The ligation product was introduced in *E. coli* DH10B cells by electroporation, and transformed bacteria were selected on LB agar medium containing 50 μg ml^−1^ kanamycin. The obtained plasmid (pBI‐RFP) was purified and used for the generation of the LYK2‐RFP construct. The full‐length *LYK2* coding sequence was amplified by PCR with a high‐fidelity DNA polymerase (Roche) from Col‐0 genomic DNA, using the following primers: CATCTCCCTTCTGAGGACCA (LYK2gFw) and GATGAGTTTAGGGCCATGATGC (LYK2gRev). The PCR product was cloned into the pGEM T‐Easy vector (Invitrogen) and used to transform *E. coli* One Shot® OmniMAX™ 2 T1R (Invitrogen) cells by electroporation. The obtained plasmid (pGEM‐LYK2) was purified and digested with KnpI and SmaI and the insert was cloned in frame upstream the RFP coding sequence in the pBI‐RFP plasmid previously digested with the same enzymes and dephosphorylated. To generate constructs for the overexpression of the untagged version of LYK2, the insert of pGEM‐LYK2 was ligated with pBI121, previously digested with KnpI and SmaI. The obtained plasmids (pBI121‐LYKL2‐RFP and pBI121‐LYK2) were introduced into *E. coli* One Shot® OmniMAX™ 2 T1R (Invitrogen) and transformed bacteria were selected on LB agar medium containing 50 μg ml^−1^ kanamycin. To generate LYK2‐GFP, the full‐length coding sequence of *LYK2* was cloned into the pDONR‐Zeo plasmid by BP cloning using a Gateway‐based system (Invitrogen). The obtained plasmid was then used for LR cloning with the destination plasmid pGWB5 (Nakagawa et al., 2007). For generation of 35S:CERK1‐GFP, 35S:CERK1‐myc and 35S:LYK5‐myc constructs for CoIP experiments, the full‐length coding sequences of *LYK5* and *CERK1* were cloned into the pDONR201 plasmid (Invitrogen) using the following primers: for *LYK5*, ggggacaagtttgtacaaaaaagcaggcttcATGGCTGCGTGTACACTCCACGCG and ggggaccactttgtacaagaaagctgggtcGTTGCCAAGAGAGCCGGAACGAAGA; for *CERK1*, ggggacaagtttgtacaaaaaagcaggcttcATGAAGCTAAAGATTTCTCTAATC and ggggaccactttgtacaagaaagctgggtcCCGGCCGGACATAAG (in lower case is indicated the sequence added to the primer for BP reaction) and then cloned into pGWB5 (GFP‐tag) and pGWB17 (myc‐tag), respectively (Nakagawa et al., 2007), using the Gateway® LR Clonase® II Enzyme Mix (Thermo Scientific). To generate constructs for BiFC experiments, total RNA was isolated from *A. thaliana* Col‐0 leaves using the Plant RNA Purification Reagent (Invitrogen) and cDNA synthesis was performed using Super script III First Strand Synthesis System (Invitrogen). The full‐length coding sequences of *LYK2* and *LYK5* were amplified by PCR using the following primers: for *LYK2*, ggggacaagtttgtacaaaaaagcaggctttATGGCTGTTTCAGTTAGTAAGC and ggggaccactttgtacaagaaagctgggtcATCTATTATACTACTCTTCTTTAC; for *LYK5*, ggggacaagtttgtacaaaaaagcaggctttATGGCTGCGTGTACACTCCA and ggggaccactttgtacaagaaagctgggtcGTTGCCAAGAGAGCCGGAA (in lower case is indicated the sequence added to the primer for BP reaction). PCR was performed using the GoTaq Long PCR Master Mix, High‐Fidelity PCR (Promega), cloned into pDONR221 (Invitrogen) and then cloned under the control of the CaMV 35S promoter in frame with the N‐ or C‐terminal half of YFP in the pC‐SpyNe‐GW and pC‐SpyCe‐GW binary vectors, respectively, as previously described (Walter et al., 2004), using the Gateway Recombination Cloning Technology (ThermoFisher Scientific). All obtained plasmids were introduced into *A. tumefaciens* strain GV3101 by electroporation. Stable transformation of Arabidopsis plants was performed by floral dip (Clough & Bent, 1998).

### 2.10 Confocal laser microscopy

An inverted laser scanning confocal microscope (LSM780 NLO; Carl Zeiss) was used for confocal analyses. For LYK2 localization, cotyledons of *lyk2‐1* 35S:LYK2‐RFP homozygous T3 seedlings or WT 35S:LYK2‐GFP T1 seedlings were analysed using 40x Zeiss plan‐neofluar/oil, 1.3 NA, DIC. RFP and GFP were detected with a 560–615 nm and a 525–550 filter set, respectively. For plasmolysis assay, samples were incubated in 0.5 M mannitol solution for 20 min. For BiFC experiments, binary vectors containing 35S:LYK2‐NYFP, 35S:LYK2‐CYFP, 35S:LYK5‐NYFP and 35S:LYK5‐CYFP were introduced into *A. tumefaciens* GV3101. *A. tumefaciens* cells were collected at OD_600_ = 0.5, and suspended in 10 mM MgCl_2_, 10 mM MES pH 5.6, and 200 μM acetosyringone. The leaf abaxial spaces of four/five‐week‐old tobacco plants were co‐infiltrated with the bacterial cell suspensions by means of a needleless syringe. Two leaves from three independently transformed plants were analysed 48 hr after infiltration. Imaging of BiFC experiments was performed using 488 nm excitation of an Argon ion laser, 25 mW. GFP was detected with a 505–530 nm filter set whereas RFP was detected with a 560–615 nm filter set. A 488/543/633 beam splitter was used for acquisition. Imaging was performed using 40x Zeiss plan‐neofluar/oil, 1.3 NA, DIC.

### 2.11 Co‐immunoprecipitation assays

*A. tumefaciens* was grown overnight in LB medium containing the appropriate antibiotics, collected by centrifugation, and then suspended in 10 mM MgCl_2_ containing 100 mM acetosyringone. After incubation at RT for at least 2 hr, the cultures were diluted to an OD_600_ = 0.5. Leaves of four‐week‐old *N. benthamiana* plants were agroinfiltrated using a needleless syringe and plants were returned to the greenhouse for 72 hr. Samples from agroinfiltrated leaves were lysed in a buffer containing 50 mM Tris (PH 7.6), 150 mM NaCl, 0.5% Triton X‐100 and protease inhibitor cocktail P9599 (Sigma, MO). Extracts were centrifuged at 14,000 × g for 15 min at 4°C. Anti‐Myc or anti‐GFP traps (Chromotek) were used for co‐immunoprecipitation experiments according to the manufacturer's instructions. Immunoblot analysis was performed as previously described (Willmann et al., 2011), using anti‐myc or anti‐GFP antibodies (Sigma‐Aldrich) at a dilution of 1:3000.

## 3 RESULTS

### 3.1 LYK2 is required for resistance to Botrytis cinerea induced by different elicitors

To investigate the role of *LYK2* in Arabidopsis immunity, two homozygous insertional lines were obtained: *lyk2‐1* (SALK_152226), carrying a predicted T‐DNA insertion in the second exon, corresponding to the extracellular domain of LYK2, and *lyk2‐2* (SALK_012441), with a predicted insertion at the very beginning of the first exon (Figure S1a,b). Untreated seedlings of both mutants showed significantly decreased levels of *LYK2* transcripts, compared to the wild type (Figure S1c). In addition, we generated two independent homozygous lines (35S:LYK2 line 1.1 and 5.15) overexpressing *LYK2* (Figure S1d). Both lines accumulated high levels of *LYK2* transcripts in the absence of any treatment (Figure S1d).

Available microarray data indicate that basal *LYK2* transcript levels are quite low in seedlings and leaves of WT plants, compared to *CERK1*, *LYK3* and *LYK5*, are similar to those of *LYK4* (Figure S2a). We examined the expression of *LYK2* in WT and mutant seedlings treated with different elicitors. Compared to water‐treated seedlings, *LYK2* transcripts in the wild type increased two‐ to four‐fold after 1 hr of treatment with OGs, flg22 or chitin (Figure S2b). In contrast, expression of *LYK2* in both *lyk2* mutants did not significantly differ after elicitation (Figure 2b). These results indicate that *lyk2‐1* and *lyk2‐2* have impaired basal and elicitor‐triggered expression of *LYK2*. Basal expression of *CERK1* and *LYK5* was comparable in WT and *lyk2* seedlings; transcripts for both genes slightly increased to a similar extent after 1 h of elicitation with flg22 or chitin, though expression levels were quite variable (Figure S2c,d). Notably, compared to the WT, *lyk2* mutants accumulated greater transcript levels of both *CERK1* and *LYK5* after 3 h of treatment with chitin, but not with flg22 (Figure S2c,d), suggesting that reduced expression of *LYK2* might trigger, in response to this MAMP, a compensatory response resulting in the enhanced expression of other *LYK* genes.

**Figure 1:**
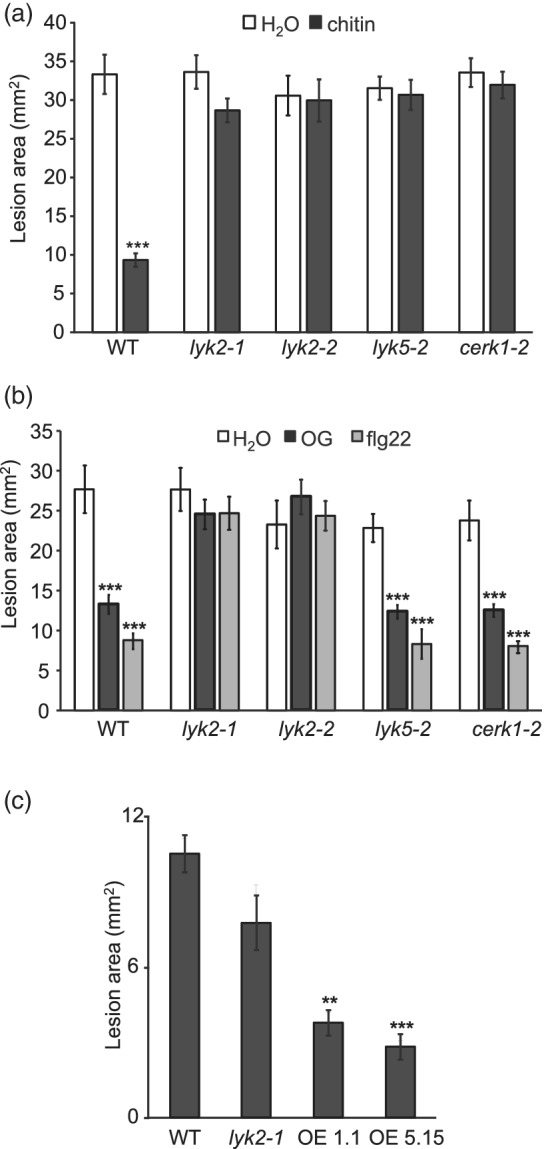
Impact of *lyk* mutations on basal and elicitor‐induced resistance to *B. cinerea*. Leaves of four‐week‐old WT, *lyk2‐1*, *lyk2‐2*, *lyk5‐2* and *cerk1‐2* plants were sprayed with water or with 100 μg ml^−1^ chitin (a), 200 μg ml^−1^ OG or 1 μM flg22 (b). After 24 h, leaves were inoculated with a *B. cinerea* spore suspension (5 × 10^5^ spores ml^−1^). (c) Leaves of four‐week‐old WT, *lyk2‐1*, 35S:LYK2 1.1 and 15.5 (OE 1.1 and OE 5.15) plants were inoculated with *B. cinerea*. Lesion areas were measured 48 hours after inoculation. Data are means ± SE (n = 12); asterisks indicate statistically significant differences between WT and mutants, according to Student's *t*‐test (**, *p* > .01; ***, *p* < .001). These experiments were repeated three times with similar results

To investigate the role of Arabidopsis LYKs in basal and chitin‐induced resistance to fungal infection, we evaluated the severity of symptoms caused by *B. cinerea* in *lyk2‐1* and *lyk2‐2*, as well as in *cerk1‐2* and *lyk5‐2*, which carry loss‐of‐function mutations in *CERK1* and *LYK5*, respectively, and are impaired in chitin perception (Cao, Liang, et al., 2014; Miya et al., 2007; Wan et al., 2008). Adult rosettes were sprayed with water or chitin and, after 24 hr, fully expanded leaves were inoculated with a *B. cinerea* spore suspension. Disease symptoms were evaluated 48 hr postinfection (hpi). None of the mutants showed increased susceptibility to *B. cinerea* after the water pre‐treatment (Figure 1a and Figure S3a,b). Pre‐treatments with chitin led to significantly smaller lesions in the wild type, but not in any of the mutants (Figure 1a and Figure S3a), indicating that induction of *Botrytis* resistance by chitin requires not only CERK1 and LYK5, but also LYK2. To test if these LYKs are also required for resistance induced by other MAMPs and DAMPs, WT and mutant plants were pre‐treated with flg22 or OGs, that can induce resistance against *B. cinerea* in Arabidopsis (Ferrari et al., 2007). Both elicitors significantly increased resistance in WT, *cerk1‐2* and *lyk5‐2* plants, but not in *lyk2‐1* and *lyk2‐2* (Figure 1b and Figure S3b). Taken together, these results suggest that CERK1, LYK2 and LYK5 are all required for chitin‐induced resistance, but only LYK2 is also necessary for resistance induced by the non‐chitin elicitors flg22 and OGs.

To test if elevated *LYK2* expression levels have an impact on resistance to *B. cinerea*, transgenic 35S:LYK2 line 1.1 and 5.15 adult plants were inoculated with the fungus. Both lines displayed significantly reduced lesions after *B. cinerea* infection (Figure 1c), indicating that high levels of expression of *LYK2* increase resistance to this pathogen.

### 3.2 LYK2 is dispensable for early chitin perception and signalling but is required for chitin‐induced callose deposition

**Figure 2:**
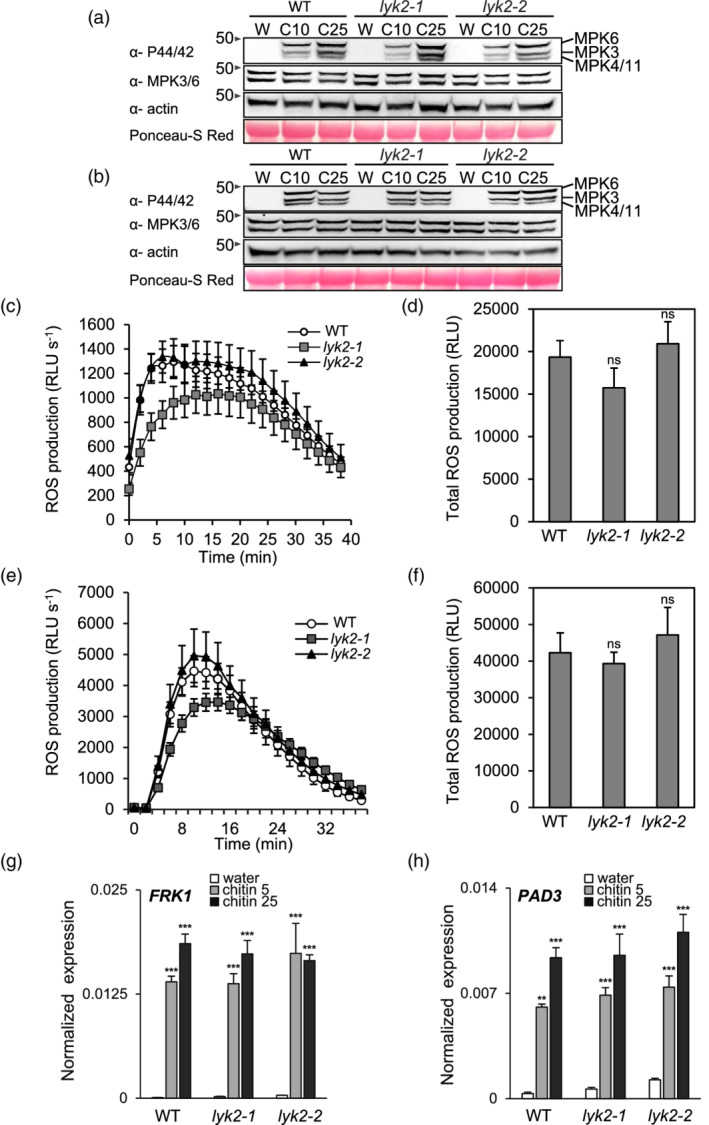
Early chitin‐induced responses are unaffected in *lyk2* mutants. (a–b) 10‐day‐old WT, *lyk2‐1* and *lyk2‐2* seedlings were treated for 5 (a) and 10 min (b) with water (W) or chitin at the concentration of 10 μg ml^−1^ (C10) or 25 μg ml^−1^ (C25). Phosphorylated MPK3, MPK4, MPK6 and MPK11 were detected by immunoblot using an anti‐P44/P42 antibody. Total MPK3 and MPK6 were detected by immunoblot using an anti‐MPK3 and anti‐MPK6 antibodies. Arrows indicate the molecular weight (in kDa) of the marker bands. Equal loading was evaluated by Ponceau‐S Red staining and using an anti‐Actin antibody. (c–f) Leaf discs of four‐week‐old WT, *lyk2‐1* and *lyk2‐2* plants were treated for the indicated times with 100 μg ml^−1^ chitin (c,d) or 100 nM flg22 (e,f). H_2_O_2_ production was measured with a luminol‐based assay and expressed in relative light units (RLU s^−1^). Data points represent the average of at least 12 discs ± SE. Bars in (d) and (f) represent the average of total H_2_O_2_ production ± SE. Differences between total RLUs in WT and *lyk2‐1* or *lyk2‐2* were not significant (ns), according to Student's *t*‐test (*p* > .05). (g, h) *FRK1* (g) and PAD3 (h) expression in WT and *lyk2* seedlings treated with water or chitin (5 and 25 μg ml^−1^) for 1 (g) and 3 h (h) was analysed by qRT‐PCR using *UBQ5* as control. Data are means ± SE (n = 3 biological replicates). Asterisks indicate statistically significant differences according to Student's *t*‐test (***, *p* < .001) These experiments were repeated three times with similar results [Colour figure can be viewed at wileyonlinelibrary.com]

**Figure 3:**
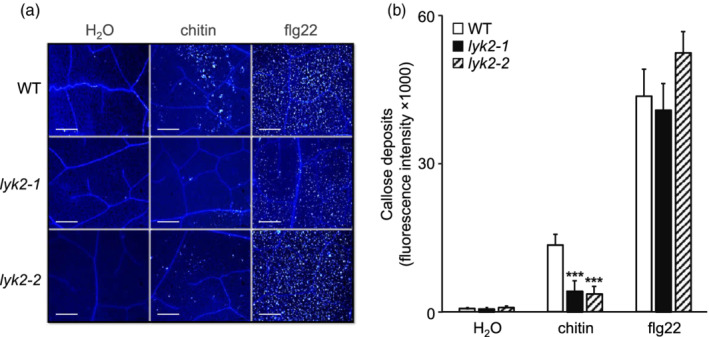
Chitin‐induced callose deposition is reduced in *lyk2* mutants. (a,b) Rosette leaves of four‐week‐old plants of WT or *lyk2‐1* and *lyk2‐2* lines were infiltrated with water, chitin (100 μg ml^−1^) or flg22 (100 nM) and stained with aniline blue 24 hr after infiltration. (a) Representative images for each treatment. Scale bars = 100 nm. (b) Callose deposits were quantified as fluorescence intensity per unit of the infiltrated leaf surface. Values represent means + SE of six different leaf samples from at least five independent plants (four microscopic fields of 0.1 mm^2^ for each leaf). Asterisks indicate statistically significant differences between mutant lines and WT according to Student's *t*‐test (***, *p* < .001). This experiment was repeated twice with similar results [Colour figure can be viewed at wileyonlinelibrary.com]

It was previously reported that *lyk2* mutants do not show defects in chitin‐induced production of ROS (Cao, Liang, et al., 2014) and expression of *WRKY53* and *MPK3* (Wan et al., 2008). It is however possible that *LYK2* regulates specific subsets of responses to this MAMP. After 5 and 10 min of treatment with two different doses of chitin (10 and 25 μg ml^−1^), phosphorylation of MPK3, MPK4 and MPK6, one of the earliest responses to this elicitor (Pitzschke & Hirt, 2009; Ramonell et al., 2005), was similar in WT, *lyk2‐1* and *lyk2‐2* seedlings (Figure 2a,b). ROS production in response to chitin and flg22 was comparable in WT, *lyk2‐1* and *lyk2‐2* seedlings (Figure 2c–f). Expression of two elicitor‐responsive marker genes, *FLG22‐INDUCED RECEPTOR‐LIKE KINASE 1* (*FRK1)* and *PHYTOALEXIN DEFICIENT 3* (*PAD3*) (Asai et al., 2002; Ferrari et al., 2007; Zhou, Tootle, & Glazebrook, 1999) in response to chitin was also unaffected in *lyk2‐1* and *lyk2‐2* seedlings, compared to the wild type (Figure 2g,h). We also analysed if a later response to chitin, namely callose deposition, requires *LYK2*. Adult rosette leaves were infiltrated with water, chitin (100 μg ml^−1^) or flg22 (100 nM) and the presence of callose was revealed by aniline blue staining. Leaves of both *lyk2* mutants, in comparison to WT plants, showed a significant reduction of the intensity and number of callose deposits in response to chitin infiltration, but not in response to flg22 (Figure 3a,b). These data suggest that LYK2 does not contribute to the initial perception of chitin and has a minor, if any, role in early signalling events that lead to MAPK activation, oxidative burst, and early gene expression, but is necessary for full activation of callose deposition, a late response to this elicitor. Moreover, *lyk2* mutants are normally responsive to flg22, though *LYK2* is required for flg22‐induced resistance to fungal infection.

### 3.3 LYK2 is required for enhanced defence responses to chitin or fungal infection in plants pre‐treated with elicitors and contributes to resistance to *Pst* DC3000

**Figure 4:**
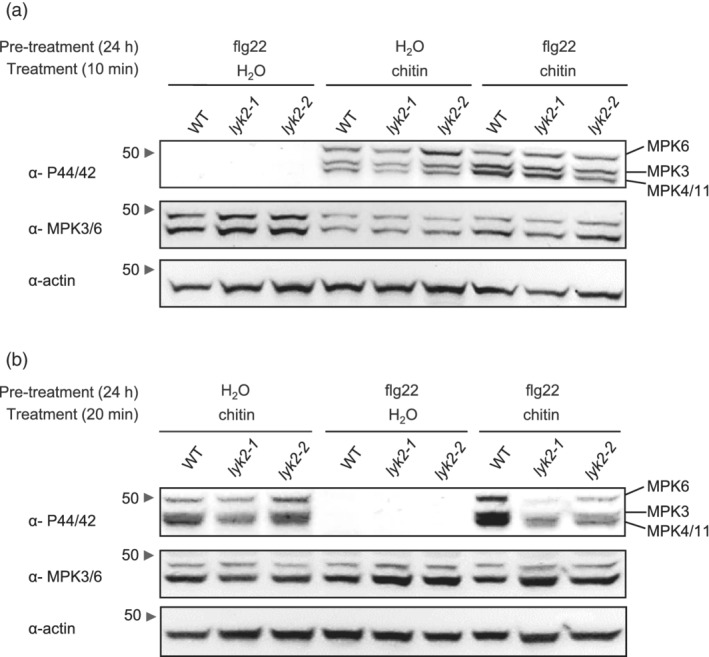
Chitin‐triggered MAPK activation in *lyk2* mutants pre‐treated with elicitors. 10‐day‐old WT, *lyk2‐1* and *lyk2‐2* seedlings were pre‐treated with water or flg22 (10 nM) for 24 hr and subsequently treated with water or chitin (25 μg ml^−1^) for 10 (a) or 20 min (b). Phosphorylated MPK3, MPK4, MPK6 and MPK11 were detected by immunoblot using an anti‐P44/P42 antibody. Total MPK3 and MPK6 were detected by immunoblot using anti‐MPK3 and anti‐MPK6 antibodies. Antibodies against actin were used as controls. The arrows indicate the molecular weight of marker bands (in kDa). This experiment was repeated three times with similar results

Basal expression of *LYK2* is very low but increases in seedlings treated with different MAMPs and DAMPs (Figure S2b). To evaluate the impact of elicitor treatments on the expression of *LYK* genes during subsequent pathogen infection, adult Arabidopsis rosette leaves were sprayed with water, OGs or flg22 and, after 24 hr, inoculated with *B. cinerea*. In control‐treated plants, *B. cinerea* infection caused a significant increase of transcripts of *CERK1* at 8 hpi and of *LYK2* and *LYK5* at 24 hpi (Figure S4a–c). Notably, plants pre‐treated with elicitors showed increased expression of these three genes even before fungal inoculation (Figure S4a–c). Moreover, *B. cinerea*‐induced up‐regulation of all three genes was faster and more robust in plants pre‐treated with flg22 or OGs (Figure S4a–c). These results suggest that induction of *LYK2* expression after elicitation might help plants respond more efficiently to chitin. To investigate this hypothesis, WT and *lyk2* seedlings were pre‐treated with water or flg22 and, after 24 h, elicited with chitin for 10 or 20 min. The pre‐treatment with flg22 did not per se result in phosphorylation of MPK3, MPK4/MPK11 and MPK6 (Figure 4a,b and Figure S5). Chitin‐induced MAPK phosphorylation in water‐pre‐treated seedlings was comparable in WT and *lyk2‐2* seedlings at both 10 and 20 min after elicitation (Figure 4a,b) and was slightly reduced in *lyk2‐1* after 20 min of elicitation (Figure 4b and Figure S5). After flg22 pre‐treatment, chitin‐induced phosphorylation at 10 min was comparable in all genotypes (Figure 4a and Figure S5a) but was strongly reduced at 20 min in *lyk2‐1* and *lyk2‐2* seedlings, compared to the wild type (Figure 4b and Figure S5b). As expected, chitin failed to induce any detectable activation of MAPKs in *cerk1‐2*, even after pre‐treatment with flg22 (Figure S5a,b). These results confirm that *LYK2* is dispensable for initial MAPK phosphorylation triggered by chitin treatment but suggest that, in plants previously exposed to an elicitor, MAPKs are dephosphorylated more rapidly if *LYK2* is not functional.

**Figure 5:**
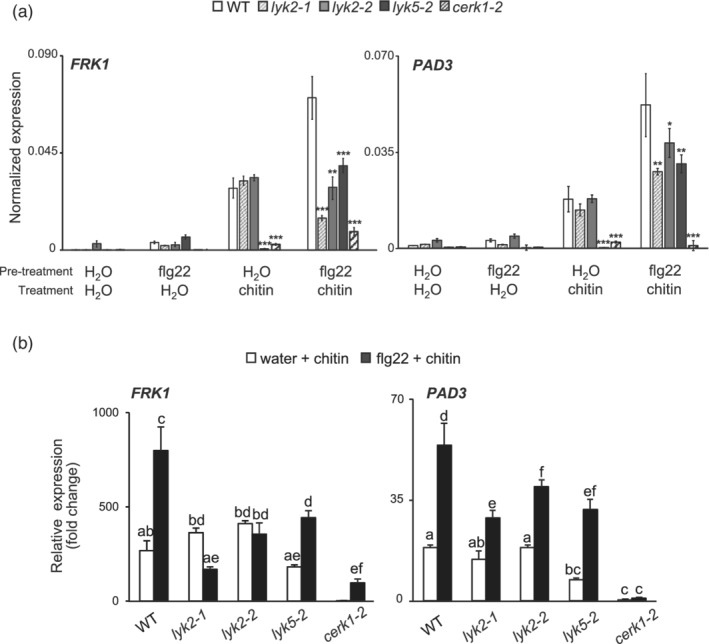
Priming of chitin‐induced gene expression in *lyk* mutants. 10‐day‐old WT, *lyk2‐1, lyk2‐2, lyk5‐2* and *cerk1‐2* seedlings were pre‐treated with water or 10 nM flg22 for 24 h and subsequently treated with water or chitin (25 μg ml^−1^) for 1 h. *FRK1* and *PAD3* expression was measured by qRT‐PCR and normalized using *UBQ5*. (a) Bars represent mean expression ± SE (n = 3 biological replicates), asterisks indicate statistically significant differences between WT and mutants, according to Student's t‐test (*, *p* < .05; **, *p* < .01; ***, *p* < .001). (b) Expression of *FRK1* and *PAD3*, relative to WT seedlings pre‐treated with water and then treated with water, of the same samples as in (a). Bars represent mean fold‐change ± SE (n = 3 biological replicates). Different letters indicate statistically significant differences, according to one‐way ANOVA followed by Tukey's HSD test (*p* < .01). This experiment was repeated three times with similar results

Pre‐treatments of WT plants with flg22 significantly enhanced chitin‐induced expression of *FRK1* and *PAD3* (Figure 5); this enhanced expression was significantly impaired in *lyk2‐1* and *lyk2‐2* plants (Figure 5). In the absence of pre‐treatments with flg22, *lyk5‐2* and *cerk1‐2* did not show induction of *PAD3* and *FRK1* in response to chitin, as expected; however, expression of both marker genes was induced by chitin in flg22‐pre‐treated *lyk5‐2* seedlings, though to a lesser extent than in the wild type, whereas *cerk1‐2* was almost completely unresponsive to chitin, even after previous exposure to flg22 (Figure 5). Taken together, the results described above confirm that *CERK1* and *LYK5*, but not *LYK2*, are required for basal responsiveness to chitin in plants and that *CERK1* is absolutely required for chitin responsiveness, and suggest that Arabidopsis, upon flg22 elicitation, acquires the ability to partially respond to chitin also in the absence of a functional LYK5. Moreover, these data indicate that LYK2 is necessary to enhance responses downstream chitin perception in plants pre‐treated with flg22.

**Figure 6:**
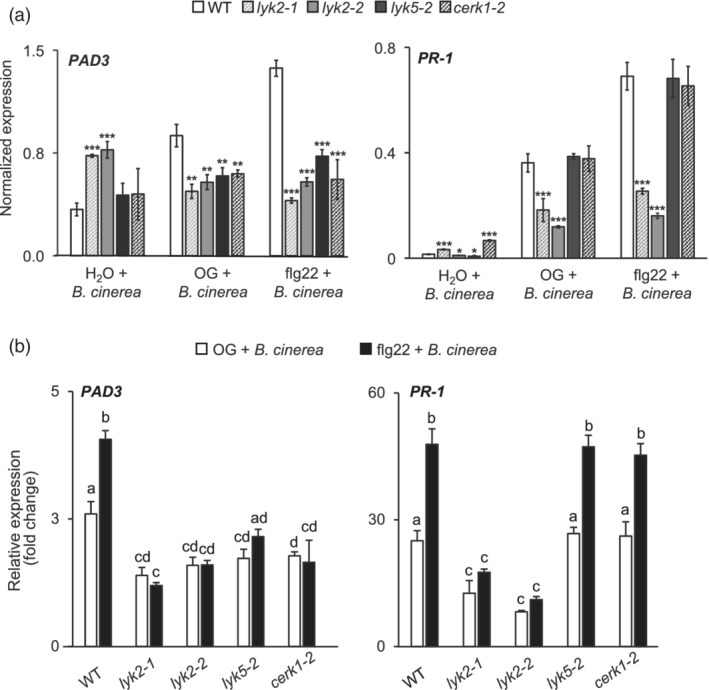
Priming of pathogen‐induced *PAD3* and *PR‐1* expression in *lyk* mutants. Four‐week‐old WT, *lyk2‐1, lyk2‐2, lyk5‐2* and *cerk1‐2* plants were sprayed with water, 200 μg ml^−1^ OG or 1 μM flg22 and inoculated after 24 hr with a *B. cinerea* spore suspension. *PAD3* and *PR‐1* expression was measured 24 hr after inoculation by qRT‐PCR and normalized using *UBQ5*. (a) Bars represent mean expression ± SE (n = 3 biological replicates); asterisks indicate statistically significant differences between WT and mutants, according to Student's t‐test (*, *p* < .05; **, *p* < .01; ***, *p* < .001). (b) Mean expression fold‐change (± SE, n = 3 biological replicates), relative to water‐treated WT plants, of the same plants as in (a). Asterisks indicate statistically significant differences between WT and mutants, according to Student's t‐test (*, *p* < .05; **, *p* < .01; ***, *p* < .001). Different letters indicate statistically significant differences, according to one‐way ANOVA followed by Tukey's HSD test (*p* < .01). This experiment was repeated three times with similar results

To further investigate the role of LYK2 in elicitor‐induced resistance, the expression of two defence‐related genes, *PAD3* and *PR‐1*, was evaluated in WT and mutant plants treated with water or flg22 and subsequently inoculated with *B. cinerea*. Camalexin production, catalysed by the cytochrome P450 CYP71B15 encoded by *PAD3* (Schuhegger et al., 2006), is necessary for elicitor‐induced resistance to *B. cinerea* (Ferrari et al., 2007) and is primed by elicitor treatments (Gravino et al., 2015; Savatin et al., 2015). *PR‐1* is a well‐known marker for SA‐dependent responses (Delaney et al., 1994; Gaffney et al., 1993), which are also important for basal resistance to *B. cinerea* (Ferrari et al., 2003) and for long‐lasting systemic acquired resistance to several pathogens (Cao, Liang, et al., 2014; Delaney et al., 1994; Gaffney et al., 1993). After 24 hr of infection, in water‐pre‐treated plants, a higher expression of *PAD3* in *lyk2* mutants, and of *PR‐1* in *lyk2‐1* and *cerk1‐2* mutants was observed, compared to the wild type, (Figure 6). In the wild type pre‐treated with flg22 or OGs, expression of *PAD3* and *PR‐1* was induced by the fungus to a greater extent than in control plants, indicating a priming effect of the elicitors (Figure 6 and Table S2), with a stronger effect observed for flg22 compared to OGs. The priming effect of the elicitors on fungal‐induced *PAD3* expression was also observed in *cerk1‐2* and *lyk5* plants, though transcript levels in these mutants reached lower levels than in the WT (Figure 6 and Table S2). In infected *lyk2* mutants, transcript levels of *PAD3* after elicitor pre‐treatments were even lower than in water‐pre‐treated plants, possibly because of the already high levels in the control, and were however comparable to those observed in *lyk5* and *cerk1‐2* (Figure 6 and Table S2). Elicitor‐primed expression of *PR‐1* during *B. cinerea* infection was observed in all tested genotypes but was strongly reduced in *lyk2‐1* and *lyk2‐2* plants, whereas it was comparable to the WT in *lyk5* and *cerk1‐2* (Figure 6 and Table S2). These data indicate that *LYK2*, *CERK1* and *LYK5* are all required for full priming of *PAD3* expression upon infection after elicitor pre‐treatments, which is likely dependent on an increased ability of the plant to respond more efficiently to chitin, whereas priming of *PR‐1* expression is dependent on *LYK2* but largely independent of chitin perception.

**Figure 7:**
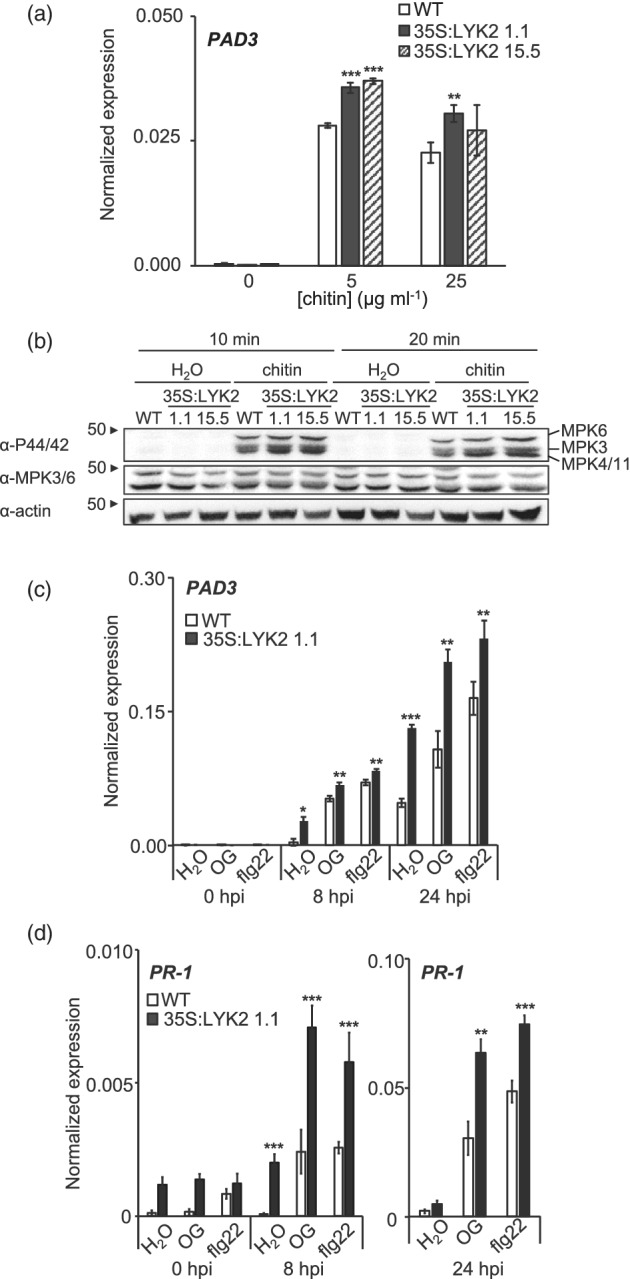
Overexpression of *LYK2* increases resistance to *B. cinerea* and expression of defence genes in responses to chitin and infection. (a) *PAD3* expression in WT, 35S:LYK2 1.1 and 15.1 seedlings, treated for 3 hr with chitin at the indicated concentrations, was determined by qRT‐PCR. *UBQ5* was used for normalization. Data are means (± SE, n = 3 biological replicates). (b) Four‐week‐old WT and 35S:LYK2 line 1.1 and line 15.5 seedlings were treated with water or chitin (25 μg ml^−1^) for the indicated time. Phosphorylated MPK3, MPK4, MPK6 and MPK11 were detected by immunoblot using an anti‐P44/P42 antibody. Total MPK3 and MPK6 were detected using anti‐MPK3 and anti‐MPK6 antibodies. Antibodies against actin were used as controls. The arrows indicate the molecular weight of marker bands (in kDa). (c, d) WT and 35S:LYK2 Line 1.1 four‐week‐old plants were sprayed with water, 200 μg ml^−1^ OG or 1 μM flg22 and inoculated after 24 hr with a *B. cinerea* spore suspension. *PAD3* (c) and *PR1* (d) expression, at the indicated times, was measured by qRT‐PCR and normalized using *UBQ5*. Data are means (± SE, n = 3 biological replicates). Asterisks indicate statistically significant differences between WT and 35S:LYK2 line 1.1 plants, according to Student's *t*‐test (*, *p* < .05; **, *p* < .01; ***, *p* < .001). The results are representative of three (a, c, and d) or two (b) independent experiments

We subsequently tested if elevated *LYK2* expression levels affect responses to chitin treatment or pathogen infection. Expression of *PAD3* in response to low doses of chitin was only moderately enhanced in transgenic seedlings overexpressing *LYK2* (Figure 7a). Consistently, MAPK activation in response to chitin was comparable in WT and 35S:LYK2 plants (Figure 7b). Notably, expression of both *PAD3* and *PR‐1* during *B. cinerea* infection was significantly increased in both water‐ and elicitor‐pre‐treated 35S:LYK2 plants (Figure 7c,d). Moreover, *PR‐1* expression was enhanced in transgenic plants even before fungal inoculation, regardless of whether they were pre‐treated with water or elicitors (Figure 7d). These data suggest that *LYK2* overexpression does not affect chitin perception or early signalling, but it can increase basal (in the case of *PR‐1*) and fungal‐induced (both *PR‐1* and *PAD3*) defence gene expression.

**Figure 8:**
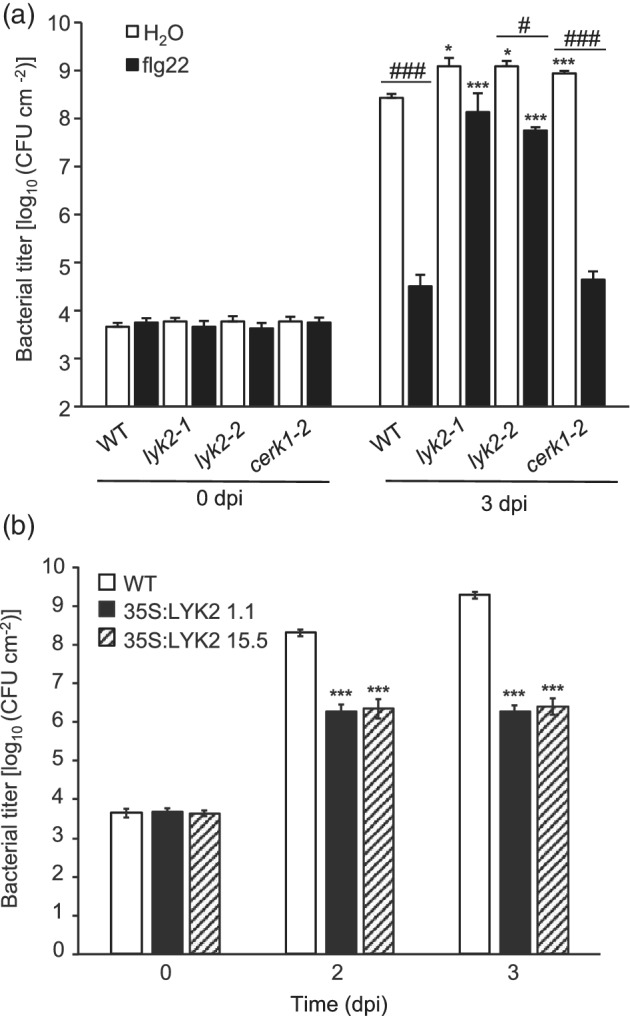
Role of LYK2 in resistance to *Pseudomonas syringae*. (a) Rosette leaves of four‐week‐old WT, *lyk2‐1*, *lyk2‐2* and *cerk1‐2* plants were sprayed with water (H_2_O) or flg22 and, after 24 hr, infiltrated with *Pst* DC3000. Bacterial growth was measured at the indicated times (days postinfection, dpi). (b) Rosette leaves of four‐week‐old WT and 35S:LYK2 lines 1.1 and 15.5 plants were infiltrated with *Pst* DC3000, and bacterial growth was measured at the indicated times. Bars indicate mean log_10_ of colony‐forming units (CFUs) per cm^−2^ (± SE, n = 12). For each time point, statistically significant differences, according to Student's *t*‐test, between similarly treated WT and mutants are indicated by asterisks (*, *p* < .05; ***, *p* < .01); in (a), differences between water‐ and flg22‐treated plants of the same genotype are indicated by pound signs (###, *p* < .01). The results are representative of three independent experiments

Since lack of *CERK1* or *LYK5* increases susceptibility to *Pst* DC3000 (Cao, Liang, et al., 2014; Gimenez‐Ibanez, Ntoukakis, & Rathjen, 2009), we tested whether altered levels of *LYK2* also affect resistance to this pathogen. Like *cerk1‐2*, Both *lyk2‐1* and *lyk2‐2* displayed increased susceptibility to *Pst* DC3000 (Figure 8a). Flg22 pre‐treatments significantly increased resistance to bacterial infection in WT and *cerk1‐2* plants, but this effect was significantly reduced in both *lyk2* mutants (Figure 8a). Consistently, plants overexpressing *LYK2* supported a reduced bacterial growth, compared to the wild type (Figure 8b). These results suggest that LYK2 contributes to both basal and elicitor‐induced resistance to bacteria.

### 3.4 LYK2 localizes in the plasma membrane and constitutively interacts with LYK5

We next investigated the subcellular localization of LYK2 and its possible interaction with other LYKs. To this aim, we generated constructs for the expression of red fluorescent protein (RFP)‐ and green fluorescent protein (GFP)‐tagged versions of the protein (35S:LYK2‐RFP and 35S:LYK2‐GFP constructs, respectively). Stable overexpression of LYK2‐RFP in *lyk2‐1* plants resulted in high basal resistance to *B. cinerea* (Figure S6a,b), as also observed in WT plants overexpressing the untagged LYK2 (Figure 1c), indicating that the tagged protein is functional. Confocal laser scanning microscopy (CLSM) of cotyledon epidermal cells of transgenic seedlings revealed a strong fluorescent signal at the plasma membrane (PM), as confirmed by plasmolysis experiments (Figure 7a). A similar pattern of localization could be observed in plants overexpressing LYK2‐GFP (Figure S7b), supporting the conclusion that LYK2 is localized in the PM. In addition, a diffuse fluorescence could occasionally be detected in the cytosol of the transgenic plants (Figure S7a,b), which might be possibly ascribed to either leakage of the protein or cleavage of the fluorescent tag.

**Figure 9:**
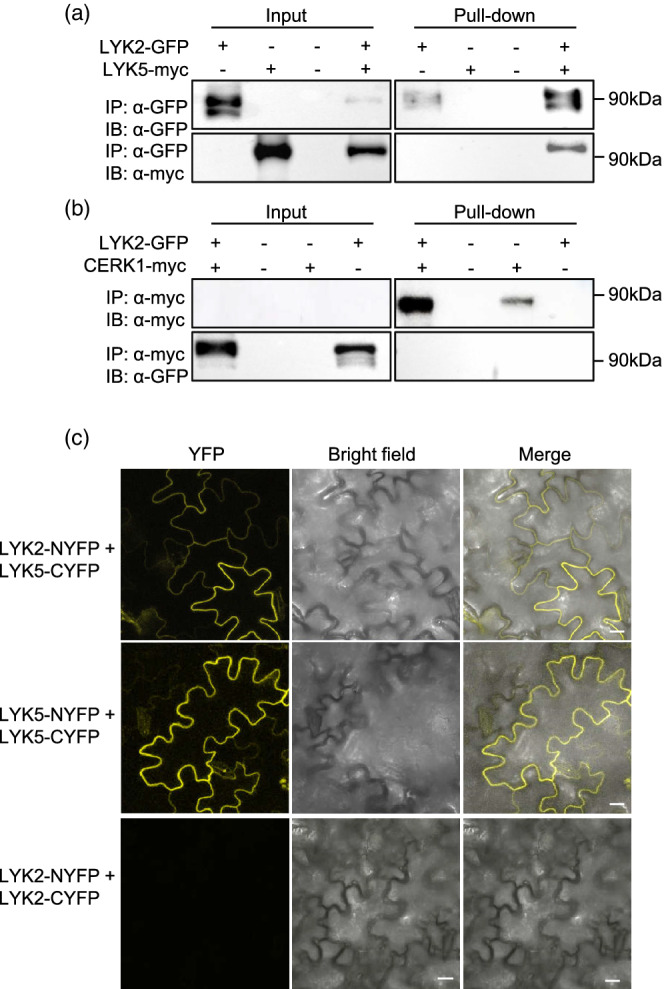
LYK2 constitutively interacts with LYK5. (a,b), LYK2‐GFP and LYK5‐myc (a) or CERK1‐myc (b) were transiently co‐expressed in *Nicotiana benthamiana*. LYK2‐GFP and CERK1‐myc were immunoprecipitated (IP) with anti‐GFP (a) and anti‐myc (b) beads, respectively, and immunoblot (IB) experiments were performed with anti‐GFP and anti‐myc antibodies. Left and right panels are cropped from the same gel. (c), LYK2 fused to the N‐terminal part of YFP (LYK2‐NYFP) and LYK5 fused to the C‐terminal part of YFP (LYK5‐CYFP) were transiently co‐expressed in *Nicotiana tabacum* by *Agrobacterium*‐mediated transformation. Images were taken 2 days after agroinfiltration by confocal laser scanning microscopy. Left panels, YFP; middle panels, bright field; right panels, merge of YFP and bright field. Bar = 20 μm [Colour figure can be viewed at wileyonlinelibrary.com]

Since LYK5 and CERK1 reside in the PM and physically interact upon chitin elicitation to induce an immune response (Cao, Liang, et al., 2014), we hypothesized that LYK2 might associate with one or both proteins. For this purpose, co‐immunoprecipitation (Co‐IP) assays were performed in *N. benthamiana* leaves transiently co‐expressing LYK2‐GFP and a myc‐tagged version of either LYK5 or CERK1 (LYK5‐myc and CERK1‐myc, respectively). Beside the expected 110 kDa band corresponding to the full‐length protein, a band of about 70 kDa, reactive to the anti‐GFP antibody, was detectable by immunoblot upon expression of LYK2‐GFP (Figure 9a), supporting the hypothesis of a partial cleavage of the cytoplasmic portion of the protein. Anti‐GFP beads could immunoprecipitate both forms of LYK2‐GFP and could also co‐purify LYK5‐myc when both proteins were co‐expressed in *N. benthamiana* (Figure 9a), suggesting that LYK2 and LYK5 can physically interact. When CERK1‐myc was transiently co‐expressed with LYK2‐GFP in *N. benthamiana*, no detectable signal for CERK1‐myc could be observed by immunoblot on total protein extracts using the anti‐myc antibody, though immunoprecipitation using anti‐myc beads revealed that the protein was indeed expressed (Figure 9b). However, under these conditions, no interaction between LYK2‐GFP and CERK1‐myc could be detected (Figure 9b).

To corroborate the hypothesis that LYK2 interacts with LYK5, in vivo bimolecular fluorescence complementation (BiFC) experiments were conducted in *N. tabacum* leaves transiently co‐expressing LYK2 and LYK5 tagged with either the N‐terminal or the C‐terminal half of the yellow fluorescent protein (NYFP and CYFP, respectively). As shown in Figure 9c, co‐transformation with 35S:LYK2‐NYFP and 35S:LYK5‐CYFP resulted in a YFP fluorescence signal, likely at the PM. As a positive control, homodimerization of LYK5 was confirmed by BiFC (Figure 9c). In contrast, no YFP fluorescence signal could be detected when LYK2‐NYFP and LYK2‐CYFP were co‐expressed (Figure 9c), suggesting that LYK2 does not homodimerize under these conditions and that the signal observed when LYK2‐NFP and LYK5‐CFP were co‐expressed was not due to non‐specific YFP reconstitution.

## 4 DISCUSSION

In this work, we have investigated the role of LYK proteins and of chitin perception in Arabidopsis basal and elicitor‐induced resistance to pathogens. Genetic evidence indicates that the contribution of chitin perception to resistance to fungi varies with different pathogens; for instance, *cerk1* mutants show increased susceptibility to *A. brassicicola* and *G. cichoracearum*, but not to *Colletotrichum higginsianum* (Miya et al., 2007; Wan et al., 2008). Here we have shown that mutants for *CERK1* or *LYK5* do not display enhanced susceptibility to *B. cinerea*. This is not unexpected, since this pathogen can release other MAMPs (Poinssot et al., 2003; Yang, Yu, Liang, Anderson, & Cao, 2018; Zhang, Qiu, Zeng, Guo, & Yang, 2015) and DAMPs (An et al., 2005; Voxeur et al., 2019), whose recognition is probably sufficient to confer a WT‐like degree of resistance also in the absence of chitin recognition. On the other hand, activation of a strong immune response occurs when plants are exposed to purified chitin, effectively protecting against subsequent *Botrytis* infections (Aziz et al., 2006). We found that, in Arabidopsis, this protection requires an intact chitin perception system, since it is abolished in *cerk1‐2* and *lyk5‐2* mutants. Unexpectedly, also *lyk2* mutants were impaired in chitin‐induced resistance, prompting us to hypothesize that *LYK2* might play a role in chitin perception and/or signalling, despite previous work failed to reveal a defect in chitin responses in mutants for this gene (Cao, Liang, et al., 2014; Wan et al., 2012). Indeed, we found that chitin‐induced MAPK phosphorylation, ROS production and early marker gene expression are largely unaffected in *lyk2* plants, whereas callose deposition, detected 24 hr after chitin treatment, is reduced in the mutants. These results suggest that *LYK2* is not important for chitin perception and early signalling under basal conditions, though it might contribute to ensure proper induction of long‐term defence responses triggered by this MAMP.

The minor role of *LYK2* in early chitin responses might be due to its low basal expression levels, since *lyk2* mutations impair only responses to this MAMP, like callose deposition and enhanced resistance to fungal infection, that are observed several hours after chitin treatment, when *LYK2* transcripts have significantly increased above basal levels. In addition, lack of *LYK2* results in greater transcript levels of *CERK1* and *LYK5* at 3 hr after chitin treatment, suggesting the existence of some compensatory mechanism that might partially mask a possible role of this gene in chitin signalling. We therefore hypothesized that increased *LYK2* expression after elicitation might increase the ability of the plant to respond to chitin, either exogenously provided or released during fungal infection. This hypothesis was initially corroborated by the observation that gene expression in response to chitin elicitation after flg22 pre‐treatment is enhanced only in WT plants, but not in plants lacking *LYK2*. Since the extracellular domain of LYK2 cannot be pulled down by chitin beads when overexpressed in protoplasts, in contrast to that of CERK1, LYK4 and LYK5 (Cao, Liang, et al., 2014), it is unlikely that LYK2 might directly bind chitin. Moreover, overexpression of LYK2 did not per se increase MAPK activation and only slightly increased gene expression induced by chitin, suggesting that this protein likely modulates responses downstream of, or independently of the early signalling events triggered by the initial activation of the chitin perception complex. On the other hand, CoIP and BiFC experiments suggest that LYK2 might physically interact with LYK5, hinting to a possible function of LYK2 in modulating the activity of the chitin perception complex. Interestingly, LYK4 also constitutively heterodimerizes with LYK5, and chitin treatments induce the formation of a tripartite complex comprising CERK1, LYK5 and LYK4 (Xue, Li, Xie, & Staehelin, 2019). LYK2, like LYK4, might act as a scaffold protein for LYK5, contributing, in primed plants, to increase the extent or duration of some responses downstream of the activation of the chitin perception complex. This hypothesis is suggested by the more rapid dephosphorylation of MAPKs in *lyk2* mutants pre‐treated with flg22. However, since *lyk2* mutants, but not *lyk5‐2* or *cerk1‐2*, are not protected against *B. cinerea* after pre‐treatments with OG or flg22 and do not show priming of *PR‐1* expression during fungal infection, LYK2 appears to mediate elicitor‐induced resistance also independently of chitin perception.

LYK2‐mediated induction of resistance against fungal infection seems to involve the regulation of multiple defence responses. We have previously observed that exogenous OGs or flg22 induce a transient increase of *PAD3* transcript levels, that return to basal levels within 12 hr of treatment (Denoux et al., 2008; Ferrari et al., 2007). However, camalexin accumulates to higher levels during *B. cinerea* infection when plants are pre‐treated with elicitors (Gravino et al., 2015), suggesting that a previous elicitation primes plants to produce this phytoalexin more rapidly upon pathogen attack. Indeed, we observed elicitor‐mediated priming of *PAD3* expression in response to fungal infection, which requires an intact chitin perception complex, beside LYK2, since it is also reduced in mutants lacking *LYK5* or *CERK1*. Therefore, in plants pre‐treated with MAMPs or DAMPs and subsequently attacked by a fungus, enhanced activation of responses triggered by chitin (or other elicitors) released during infection might increase *PAD3* expression and camalexin accumulation, enhancing resistance. We have previously observed that the Arabidopsis *bak1‐5* mutant, which is strongly impaired in flg22‐induced responses (Roux et al., 2011; Schwessinger et al., 2011), is also compromised in both basal and flg22‐induced resistance to *B. cinerea* (Gravino et al., 2017). Notably, *BAK1* is required for responses to different MAMPs, including flg22, PGN and lipopolysaccharides, but not to chitin (Shan et al., 2008), confirming that chitin perception per se is not necessary for basal resistance to the fungus. It was subsequently reported that co‐inoculation of *B. cinerea* with flg22 results in increased resistance to the fungus, and that this resistance depends on the BAK1‐mediated phosphorylation of the juxtamembrane domain of CERK1, which in turn increases sensitivity to chitin (Gong et al., 2019). Flg22‐induced priming of *PAD3* expression requires both LYK2 and an intact chitin perception system, comprising CERK1 and LYK5. The lack of evidence for a role of LYK2 in direct chitin perception, and the observation that *LYK2*, but not *CERK1* or *LYK5*, is required for enhanced resistance to *B. cinerea* and for priming of *PR‐1* expression when plants are pre‐treated with elicitors 24 hr before inoculation indicate that LYK2 regulates, upon elicitation, a long‐lasting ability of the plant to prime some defence responses, beside *PAD3* expression, independently of chitin perception. Interestingly, mutations in *LYK2* reduce basal resistance to *Pst* DC3000, similar to what observed for *cerk1‐2*, but, in contrast to the latter, also strongly impair flg22‐induced resistance against this pathogen. This is in agreement with the hypothesis that LYK2 might positively regulate long‐term defence responses downstream of recognition of different elicitors, though it cannot be ruled out that it might directly participate to PGN perception/signalling, as previously demonstrated for CERK1 and OsCERK1 (Ao et al., 2014; Buist, Steen, Kok, & Kuipers, 2008; Gust, Willmann, Desaki, Grabherr, & Nurnberger, 2012; Willmann et al., 2011). The PM‐associated Ca^2+^‐binding protein PCaP1 was recently shown to mediate OG‐ and flg22‐induced resistance to *B. cinerea* and to be required for elicitor‐triggered priming of defence gene expression during infection (Giovannoni et al., 2021). Further investigation is however required to determine whether LYK2 and PCaP1 act in the same pathway.

Modulation of responses triggered by MAMPs and DAMPs is crucial to ensure proper protection against invading pathogens without excessive cost in terms of growth. In this context, LYK2 appears to be a key regulator of priming of defence responses, ensuring that plants display enhanced resistance after pre‐exposure to an elicitor. Previous work suggests that LYK3, another Arabidopsis LYK, exerts an opposite role, negatively regulating defence responses, as lack of a functional protein causes constitutive expression of defence responses, including *PAD3* expression, and increased resistance to *B. cinerea* (Paparella et al., 2014). The negative role of LYK3 in immunity might be mediated by the phytohormone abscisic acid (ABA), as this protein is required for both ABA‐mediated repression of elicitor responses and for some physiological responses to this hormone (Paparella et al., 2014). Interestingly, loss of LYK3 also results in increased sensitivity to salt stress, suggesting that suppression of defence responses might contribute to properly counteract abiotic stresses (Paparella et al., 2014). Increasing evidence indeed suggests that CERK1 might regulate responses to abiotic stresses, as *cerk1* mutants also show increased sensitivity to salt stress (Espinoza, Liang, & Stacey, 2017), and overexpression of a fungal chitinase causes increased tolerance to salt in a CERK1‐dependent manner (Brotman et al., 2012). These observations lead to speculate that LYK proteins might have a more general function in balancing responses against different stresses beyond their role in MAMP perception. The possible function of LYK2 and other LYKs in modulating responses to different stresses therefore deserves future investigation.

The ability to mount stronger defence responses after a previous exposure to a MAMP or DAMPs might avoid overactivation of plant immunity, not only preventing growth‐defence trade‐offs that might be triggered by the numerous microorganisms present in the environment (Yu, Pieterse, Bakker, & Berendsen, 2019), but possibly favouring the interaction with beneficial microorganisms. Increasing evidence indicates that LYKs play multiple functions in plant immunity and symbiosis. For instance, OsCERK1 is required for mycorrhizal colonization (Miyata et al., 2014; Zhang et al., 2015), and its homologs in *L. japonicus* (LjLYS6) and *M. truncatula* (MtLYK9) also play a dual role in immunity and symbiosis (Bozsoki et al., 2017; Gibelin‐Viala et al., 2019). Notably, rhizobial LCOs inhibit flg22 responses in Arabidopsis in a LYK3‐dependent manner (Liang et al., 2013), and *M. truncatula* mutants for MtLYK9 are less colonized by the arbuscular mycorrhiza *Rhizophagus irregularis* but are more susceptible to the oomycete *Aphanomyces euteiches* (Gibelin‐Viala et al., 2019). Future work will help determine if LYK2 is involved in the fine‐tuning of plant responses to different microorganisms.

In conclusion, our results indicate that LYK2 has a limited role in chitin perception, but it is necessary to ensure a robust and durable resistance to pathogens after elicitor pre‐treatments, priming activation of defence responses downstream of and/or independently of chitin perception. Future studies will help elucidate the molecular mechanism of action of LYK2, providing novel clues about how plants modulate immunity. This knowledge might be important not only to improve crop resistance to microbial diseases, but also to increase our understanding of the complexity of the interactions between plants and microbes in the environment.

## CONFLICT OF INTEREST

The authors declare no conflicts of interest.

## AUTHOR CONTRIBUTION

Moira Giovannoni, Damiano Lironi, Chiara Paparella, Lucia Marti, Valeria Vecchi, Andrea A. Gust and Simone Ferrari designed and performed experiments and analysed data. Moira Giovannoni, Damiano Lironi and Simone Ferrari wrote the paper. Thorsten Nürnberger, Valeria Vecchi, Andrea A. Gust and Giulia De Lorenzo contributed to revise the final version of the manuscript. All authors read and approved the final manuscript.

## Supporting information


**Figure S1** Characterization of *lyk2* insertional mutant lines and of plants overexpressing LYK2.
**Figure S2**. Basal and elicitor‐induced expression of *LYK2* in WT and *lyk2* plants.
**Figure S3**. Lesion development in *lyk* mutants infected with *Botrytis cinerea* after pre‐treatment with elicitors.
**Figure S4**. Basal and primed expression of *LYK* genes during fungal infection.
**Figure S5**. Chitin‐triggered MAPK activation in *lyk2‐1* and *cerk1‐2* mutants.
**Figure S6**. Overexpression of LYK2‐RFP increases resistance to *B. cinerea*.
**Figure S7**. LYK2 localizes at the plasma membrane.

**Table S1** Primers used for qRT‐PCR.
**Table S2**. Statistical comparisons of defence gene expression in water‐ and elicitor‐treated WT and mutant plants inoculated with *B. cinerea*.

## Data Availability

All data generated or analysed during this study are included in this published article and its supplementary information files.
