# Supplementary material for: The Arabidopsis thaliana LysM‐containing Receptor‐Like Kinase 2 is required for elicitor‐induced resistance to pathogens
Source: Plant Cell Environ. 2021 Sep 30;44(12):3775–92. doi: 10.1111/pce.14192 (PMC9293440; doi:10.1111/pce.14192)
Supplement: Supplementary file 1 — Figure S1 Characterization of lyk2 insertional mutant lines and of plants overexpressing LYK2. Figure S2. Basal and elicitor‐induced expression of LYK2 in WT and lyk2 plants. Figure S3. Lesion development in lyk mutants infected with Botrytis cinerea after pre‐treatment with elicitors. Figure S4. Basal and primed expression of LYK genes during fungal infection. Figure S5. Chitin‐triggered MAPK activation in lyk2‐1 and cerk1‐2 mutants. Figure S6. Overexpression of LYK2‐RFP increases resistance to B. cinerea. Figure S7. LYK2 localizes at the plasma membrane. [file PCE-44-3775-s002.pdf]

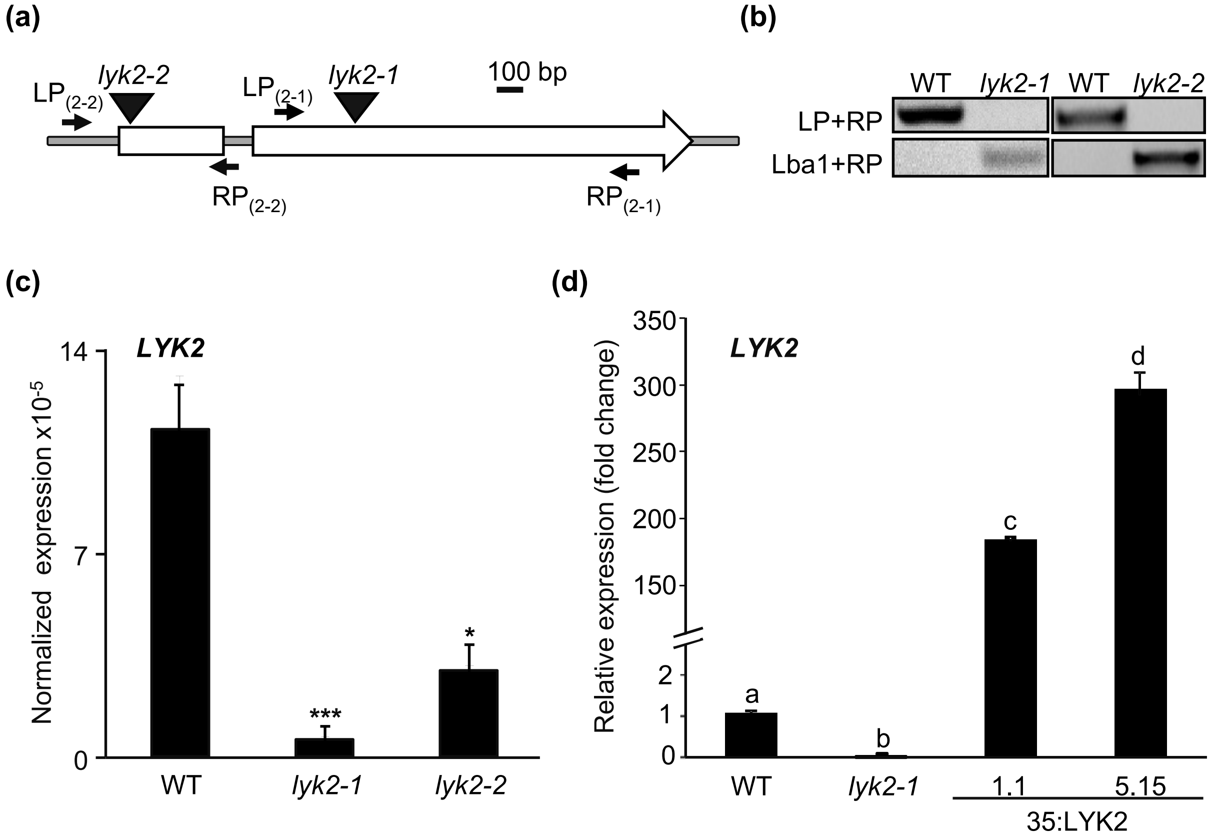

Supplementary Figure 1. Characterization of *lyk2* insertional mutant lines and of plants overexpressing LYK2. (a) Schematic representation of the T-DNA insertions in the *lyk2-1* and *lyk2-2* mutants. Exons are represented by white blocks. The triangles indicate the T-DNA insertions. Arrows indicate the position of the primers used for genotyping, namely $LP_{(2-1)}$ and $RP_{(2-1)}$ for *lyk2-1* and $LP_{(2-2)}$ and $RP_{(2-2)}$ for *lyk2-2.* **(b)** Genomic DNA from WT, *lyk2-1* and *lyk2-2* homozygous plants was subjected to PCR using the primer pairs specific for the WT gene (LP+RP) or for the T-DNA insertion (Lba1+RP). Primer sequences are listed in Suppl. Table 1. **(c)** Accumulation of *LYK2* transcripts was analysed by qRT-PCR in ten-day-old WT, *lyk2-1* and *lyk2-2* seedlings, using *UBQ5* as reference gene. Bars represent mean expression (± SD, n = 3 independent replicates). Asterisks indicate significant differences between WT and mutants, according to Student's *t*-test (***, P < 0.001; *, P < 0.05). **(d)** *LYK2* transcript levels in ten-day-old untransformed WT and *lyk2-1* seedlings and in two independent lines transformed with 35S:LYK2 (line 1.1 and 5.15) were quantified as in **(c)**. Bars represent mean relative expression, compared to the WT (± SD, n = 3 independent replicates). Different letters indicate statistically significant differences, according to one-way ANOVA followed by Tukey's HSD test (P < 0.01). The results are representative of two independent experiments.

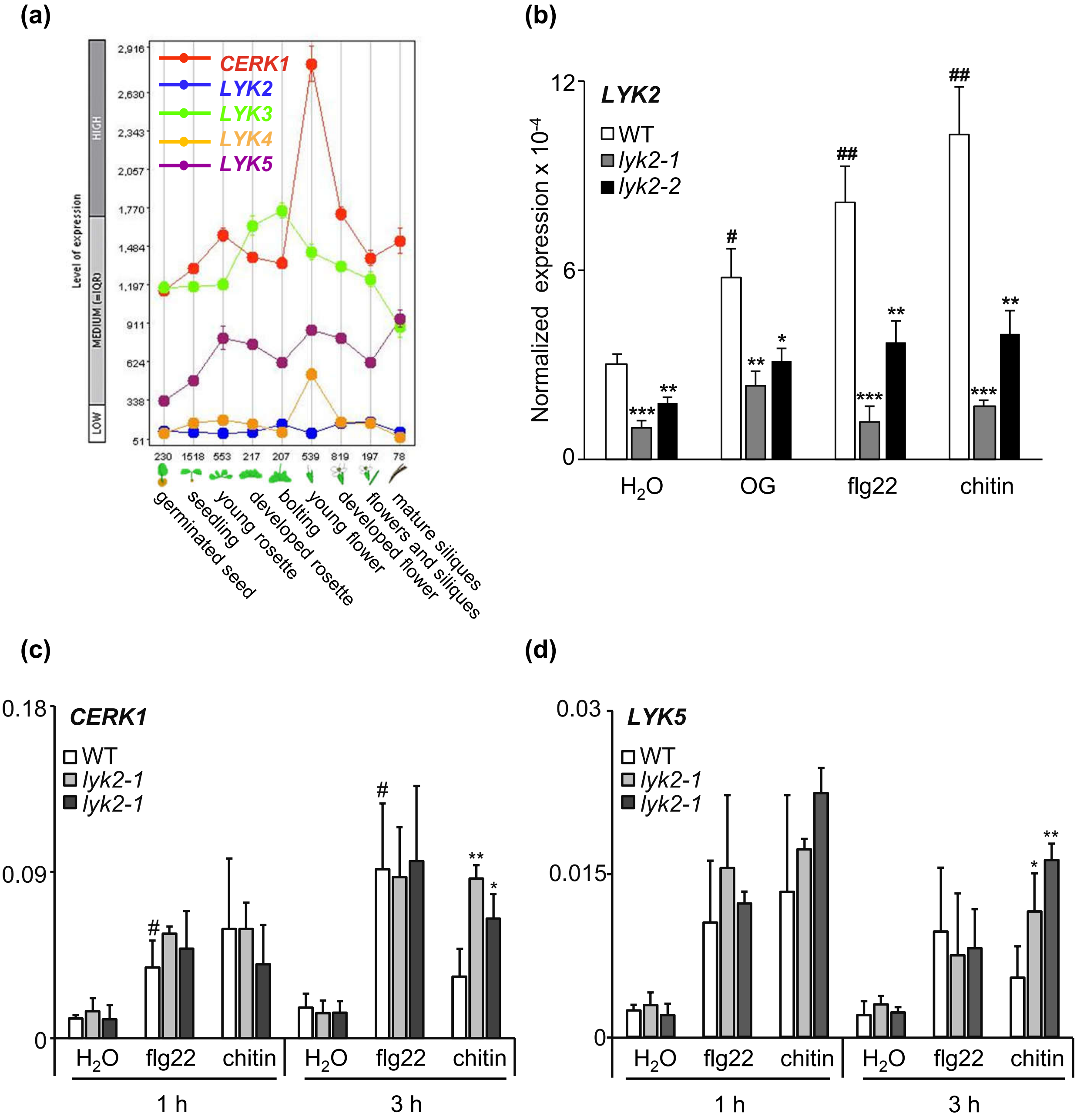

Supplementary Figure 2. Basal and elicitor-induced expression of *LYK2* in WT and *lyk2* plants. (a) Developmental stage-specific expression levels of *LYK2*, *CERK1, LYK3*, *LYK4* and *LYK5* were obtained from publicly available data using Genevestigator. **(b-d)** Ten-day-old WT, *lyk2-1* and *lyk2-2* seedlings were treated with OGs (50 µg ml^{-1}) **(b)**, flg22 (10 nM), chitin (25 µg ml^{-1}), or water as control **(b-d)**, and accumulation of *LYK2* **(b)**, *CERK1* **(c)** and *LYK5* **(d)** transcripts after 1 h **(b-d)** and 3 h **(c-d)** was analysed by qRT-PCR. *UBQ5* was used as reference gene. Bars indicate mean expression ± SE (n = 3 independent replicates). Asterisks indicate significant differences between WT and mutants, according to Student's *t*-test (*** , P < 0.001; **, P < 0.01; *, P < 0.05). For each genotype, pound signs indicate significant differences between elicitor- and water-treated plants, according to Student's *t*-test (#, P < 0.05). This experiment was repeated twice with similar results.

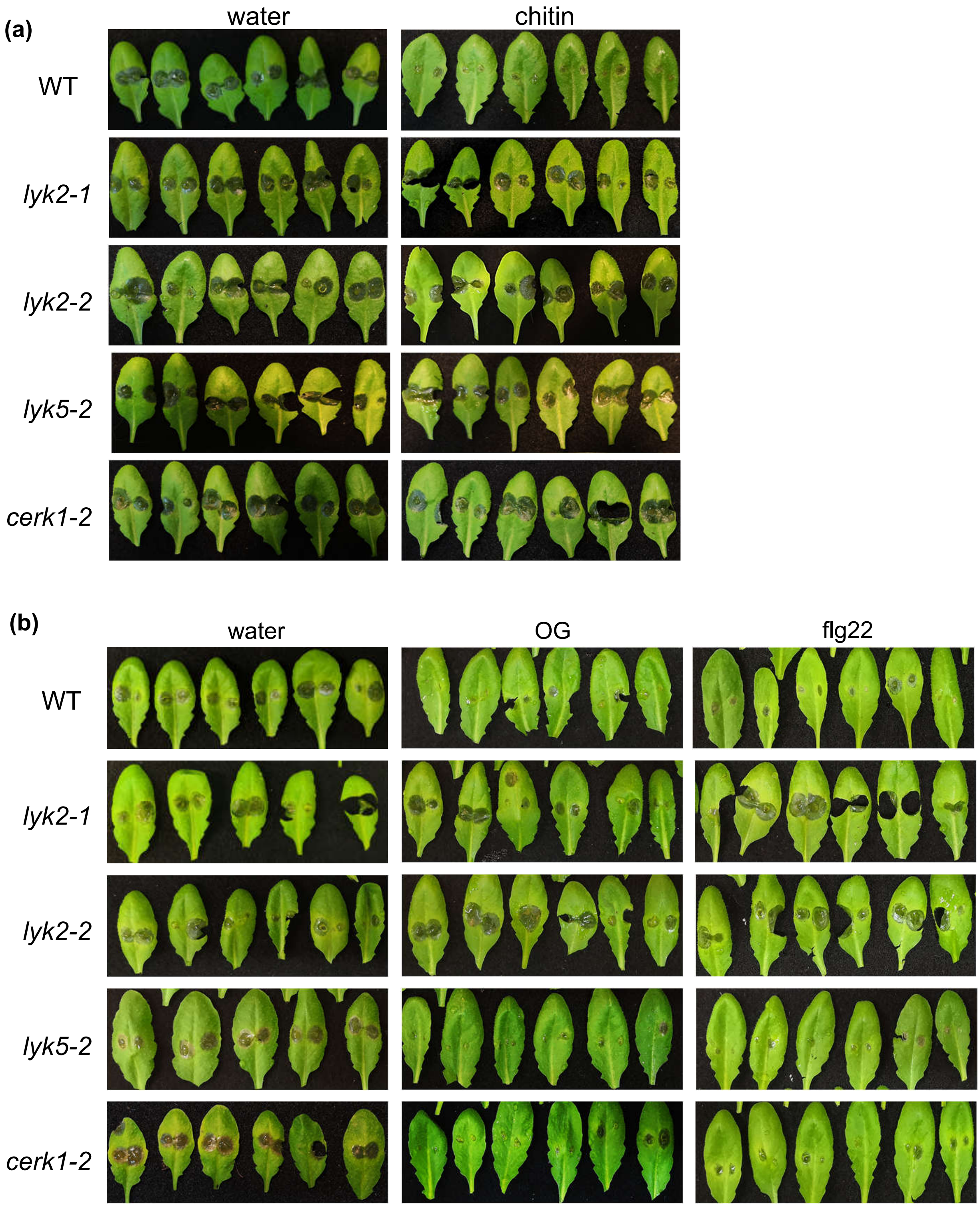

Supplementary Figure 3. Lesion development in *lyk* mutants infected with *Botrytis cinerea* after pre-treatment with elicitors. Leaves of four-week-old WT, *lyk2-1*, *lyk2-2*, *lyk5-2,* and *cerk1-2* plants were sprayed with water or 100 µg ml^{-1} chitin **(a)**, 200 µg ml^{-1} OG or 1 µM flg22 **(b)**. After 24 h, leaves were inoculated with a *B. cinerea* spore suspension. Representative images of infected leaves 48 h after inoculation are shown. This experiment was repeated three times with similar results.

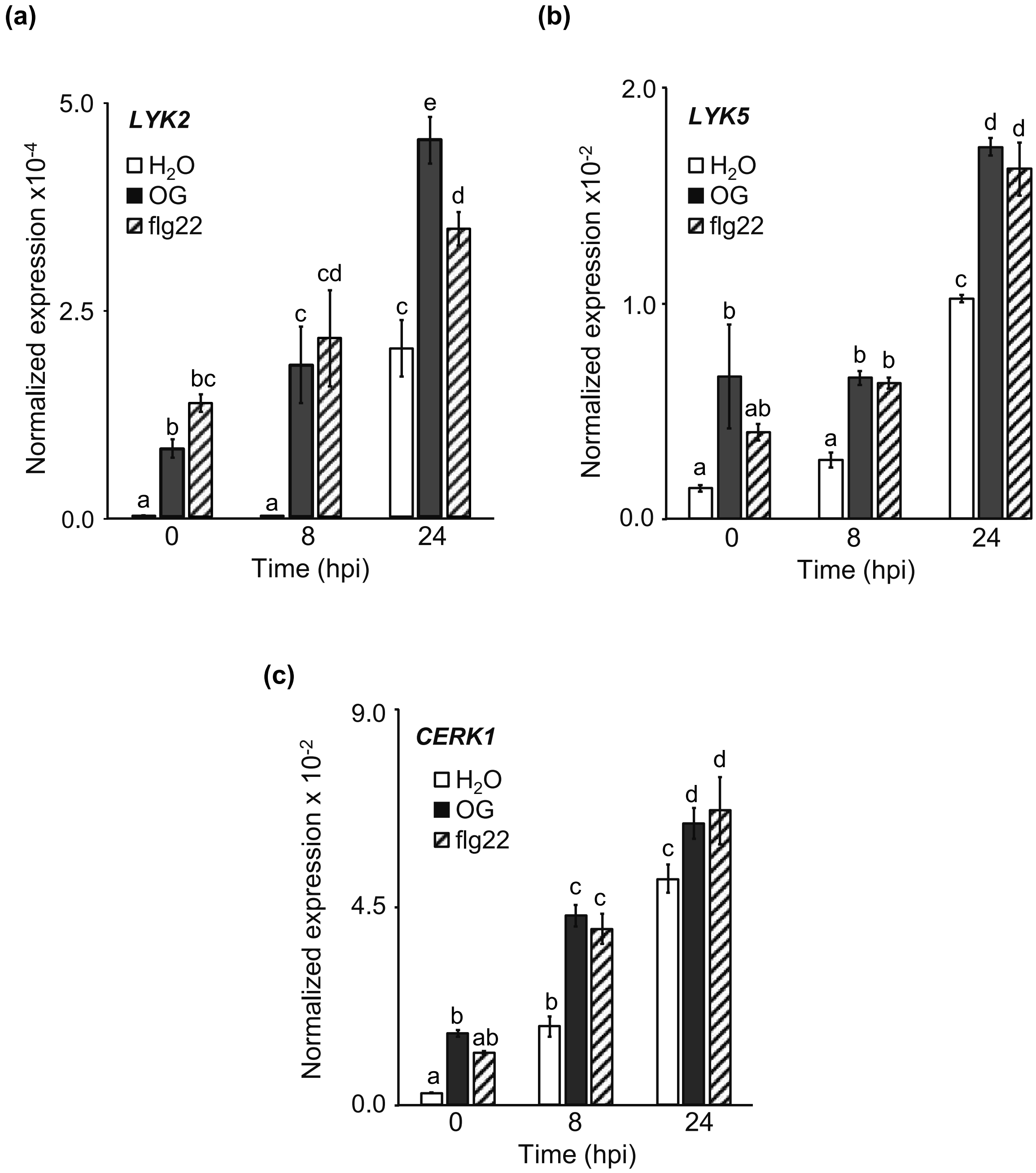

Supplementary Figure 4. Basal and primed expression of *LYK* genes during fungal infection. Four-week-old Arabidopsis leaves were treated with water, OGs (200 µg ml^{-1}) or flg22 (1 µM) and, after 24 h, they were inoculated with *B. cinerea*. Accumulation of *LYK2* **(a)**, *LYK5* **(b)** and *CERK1* **(c)** transcripts was analysed at 0, 8, and 24 h post-inoculation (hpi) by qRT-PCR. *UBQ5* was used for normalization. Bars indicate average expression of three technical replicates. Different letters indicate statistically significant differences according to one-way ANOVA followed by Tukey's HSD test ($P < 0.05$). This experiment was repeated twice with similar results.

(a)

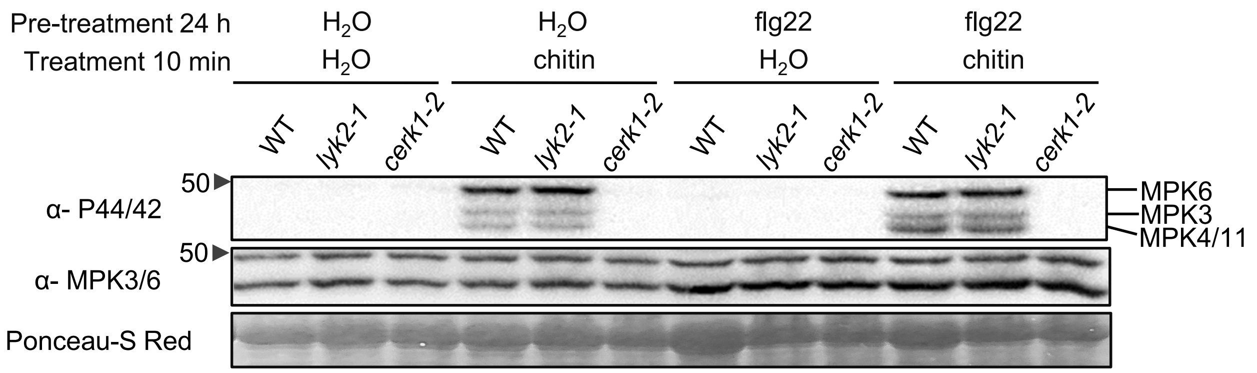

(b)

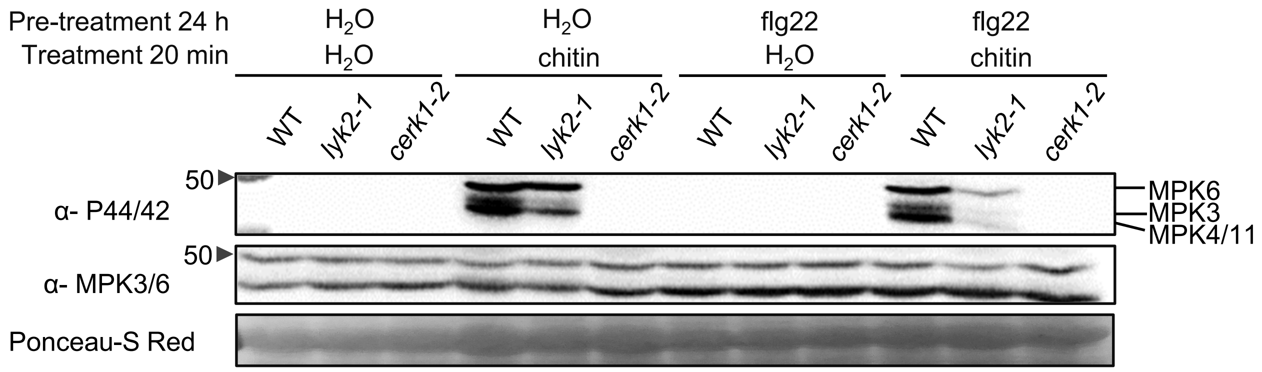

Supplementary Figure 5. Chitin-triggered MAPK activation in *lyk2-1* and *cerk1-2* mutants. Ten-day-old WT, *lyk2-1* and *cerk1-2* seedlings were pre-treated with water or flg22 (10 nM) for 24 h and subsequently treated with water or chitin (25 µg ml^{-1}) for 10 min **(a)** or 20 min **(b)**. Phosphorylated MPK3, MPK4, MPK6 and MPK11 were detected by immunoblot using an anti-P44/P42 antibody. Antibodies against total MPK3 and MPK6 were used as controls. Equal loading was evaluated by Ponceau-S Red staining. Arrows indicate the molecular weight (in kDa) of the marker bands.

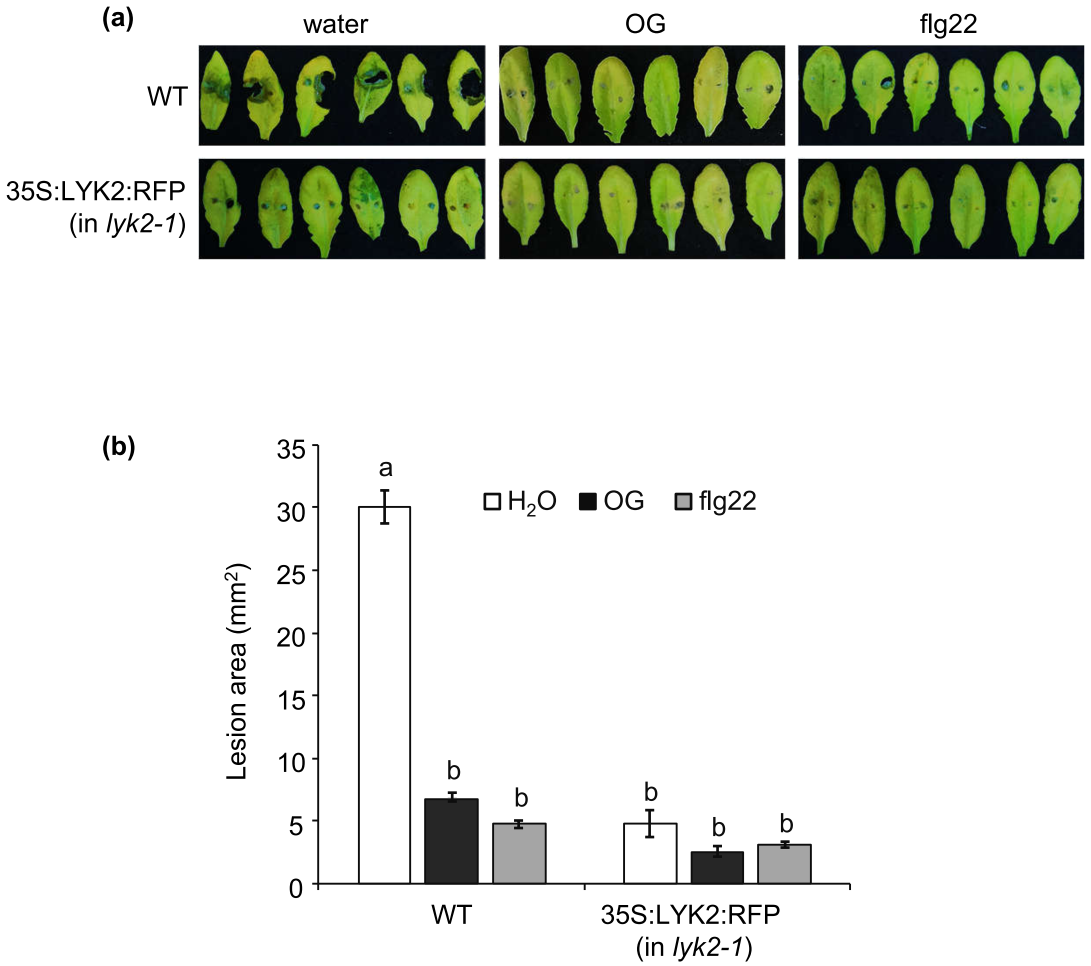

Supplementary Figure 6. Overexpression of LYK2-RFP increases resistance to *B. cinerea*. (a-b) Leaves of WT and *lyk2-1* plants transformed with 35S:LYK2-RFP were treated with water, OGs (200 µg ml^{-1}) or flg22 (1 µM) and inoculated with *B. cinerea* 24 h after elicitor treatment. **(a)** Representative images of infected leaves at 48 h post infection. **(b)** Average lesion area (± SE, n = 12). Different letters indicate statistically significant differences according to one-way ANOVA followed by Tukey's HSD test ($P < 0.05$). This experiment was repeated twice with similar results.

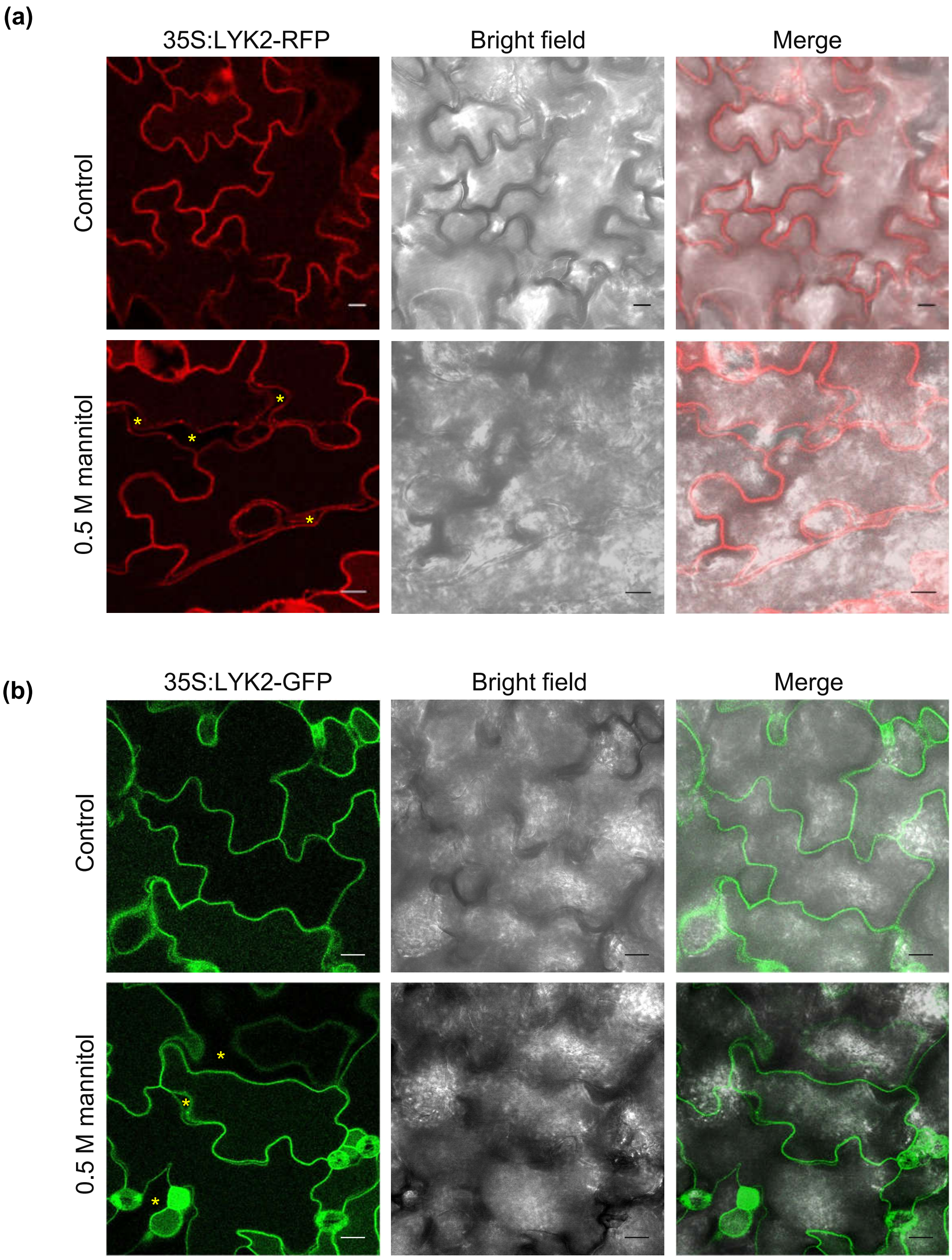

Supplementary Figure 7. LYK2 localizes at the plasma membrane. Representative confocal laser scanning microscopy, bright field and merge images of epidermal cells of cotyledons of transgenic Arabidopsis *lyk2-1* plants transformed with 35S:LYK2-RFP **(a)** and of Arabidopsis WT plants transformed with 35S:LYK2-GFP **(b),** treated with water or 0.5 M mannitol for 20 min. The images are of single focal planes. Bars = 10 µm.
