# Supplementary material for: The Arabidopsis thaliana LysM‐containing Receptor‐Like Kinase 2 is required for elicitor‐induced resistance to pathogens
Source: Plant Cell Environ. 2021 Sep 30;44(12):3775–92. doi: 10.1111/pce.14192 (PMC9293440; doi:10.1111/pce.14192)
Supplement: Supplementary file 2 — Table S1 Primers used for qRT‐PCR. Table S2. Statistical comparisons of defence gene expression in water‐ and elicitor‐treated WT and mutant plants inoculated with B. cinerea. [file PCE-44-3775-s001.docx]

**Supplementary Table 1. Primers used for qRT-PCR.**

| **GENE** | **AGI CODE** | **FORWARD PRIMER (5’-3’)** | **REVERSE PRIMER (5’-3’)** |
| --- | --- | --- | --- |
| ***LYK2*** | **AT3G01840** | AAGCTGAGGGAAGTGATGGA | TCGTCATCCACCAATCTTGA |
| ***CERK1*** | **AT3G21630** | TCGAAGGGTGATTCGTTTT | CCACCTTGCCCAATCTTAAA |
| ***LYK5*** | **AT2G33580** | CTCAAACGCCAGTTGATCCT | CAACGACGACGGTAATGACTT |
| ***UBQ5*** | **AT3G62250** | GGAAGAAGAAGACTTACACC | AGTCCACACTTACCACAGTA |
| ***FRK1*** | **AT2G19190** | TTAAACTCGACGATGCAACA | GATGGAAGTTTTCCCGTTTT |
| ***PAD3*** | **AT3G26830** | TCGCTGGCATAACACTATGG | TTGGGAGCAAGAGTGGAGT |
| ***PR1*** | **AT2G14610** | GGGAAAACTTAGCCTGGGGT | GCACATCCGAGTCTCACTGA |

**Supplementary Table S2. Statistical comparisons of defense gene expression in water- and elicitor-treated WT and mutant plants inoculated with *B. cinerea*.** With reference to Fig. 6a, the P value of the statistical analysis of the differences between water- and elicitor-treated plants, according to Student’s t-test, are indicated for each genotype, (*, P < 0.05; **, P < 0.01; ***, P < 0.001).

| ***PAD3*** | WT | *lyk2-1* | *lyk2-2* | *lyk5-2* | *cerk1-2* |
| --- | --- | --- | --- | --- | --- |
| OG + *B.cinerea* | 0.00057 *** | 0.00108 ** | 0.00701 ** | 0.08400 | 0.1783 |
| flg22 + *B.cinerea* | 1.81E-05 *** | 1.42E-05 *** | 0.00374 ** | 0.0079 ** | 0.6435 |

| ***PR1*** | WT | *lyk2-1* | *lyk2-2* | *lyk5-2* | *cerk1-2* |
| --- | --- | --- | --- | --- | --- |
| OG + *B.cinerea* | 6.33E-05 *** | 0.00388 ** | 2.43E-06 *** | 6.65E-06 *** | 0.0003 ** |
| flg22 + *B.cinerea* | 2.45E-05 *** | 4.19E-06 *** | 1.62E-05 *** | 7.72E-06 *** | 1.45E-05*** |
